# Adaptive continual botnet detection with self-supervised prototypical networks and few-shot learning for evolving botnet detection

**DOI:** 10.3389/frai.2026.1865639

**Published:** 2026-08-24

**Authors:** Niranjana Junar, Karmel Arockiasamy

**Affiliations:** School of Computer Science and Engineering, Vellore Institute of Technology, Chennai, India

**Keywords:** cyber security, deep learning, IoT botnets, IoT networks, zero-day attacks

## Abstract

**Introduction:**

One of the problems faced by botnet intrusion detection systems is the detection of unknown attacks or zero-day attacks. Zero-day attacks give security analysts very little time for action since there is only little information that is known about the new attack that is launched, given very few samples available for the training process. Class imbalance issues faced by many benchmark datasets also reduce the ability to detect a zero-day attack. It leads to very few samples of a particular class, which makes it difficult to trace different types of the same attack. The rapid evolution of botnets also poses a challenge to the existing models, which often fail to adapt to the new attack patterns without catastrophic forgetting of previously learned knowledge.

**Methods:**

The objective of this study was to develop a model for adaptive botnet detection in cases of zero-day attacks and imbalanced dataset availability. The study integrates self-supervised learning (SSL), enhanced prototypical networks with attention mechanisms, and few-shot learning (FSL) to overcome the existing issues. Wasserstein generative adversarial network with gradient penalty (WGAN-GP) generates synthetic samples to handle class imbalance. An adaptive continual learning module is used, which combines elastic weight consolidation (EWC) and an experience replay buffer which enables the model to accommodate new botnet behaviors while preserving performance on previously seen samples. Moreover, a drift detector using the KS test, Wasserstein distance, and class-prior shift triggers model adaptation when concept drift is identified.

**Results:**

The performance of the proposed model was evaluated on the CIC IoT dataset 2023, which demonstrated an exceptional accuracy of 98.50%, outperforming the traditional baselines. Few-shot learning evaluations show strong generalization with as few as 1–5 shots per class. This result shows the overall achievement of the model in zero-day attack detection and real-time adaptation to the evolving botnet threats.

**Discussion:**

The findings demonstrate that the proposed framework provides an effective approach for zero-day botnet attack detection while maintaining knowledge of previously observed attacks. The combination of few-shot learning and adaptive continual learning improves the model's ability to respond to emerging botnet behaviours without requiring extensive retraining. Thus, the proposed approach shows potential for adaptive and real-time botnet detection in evolving IoT network environments.

## 1 Introduction

**Figure 1:**
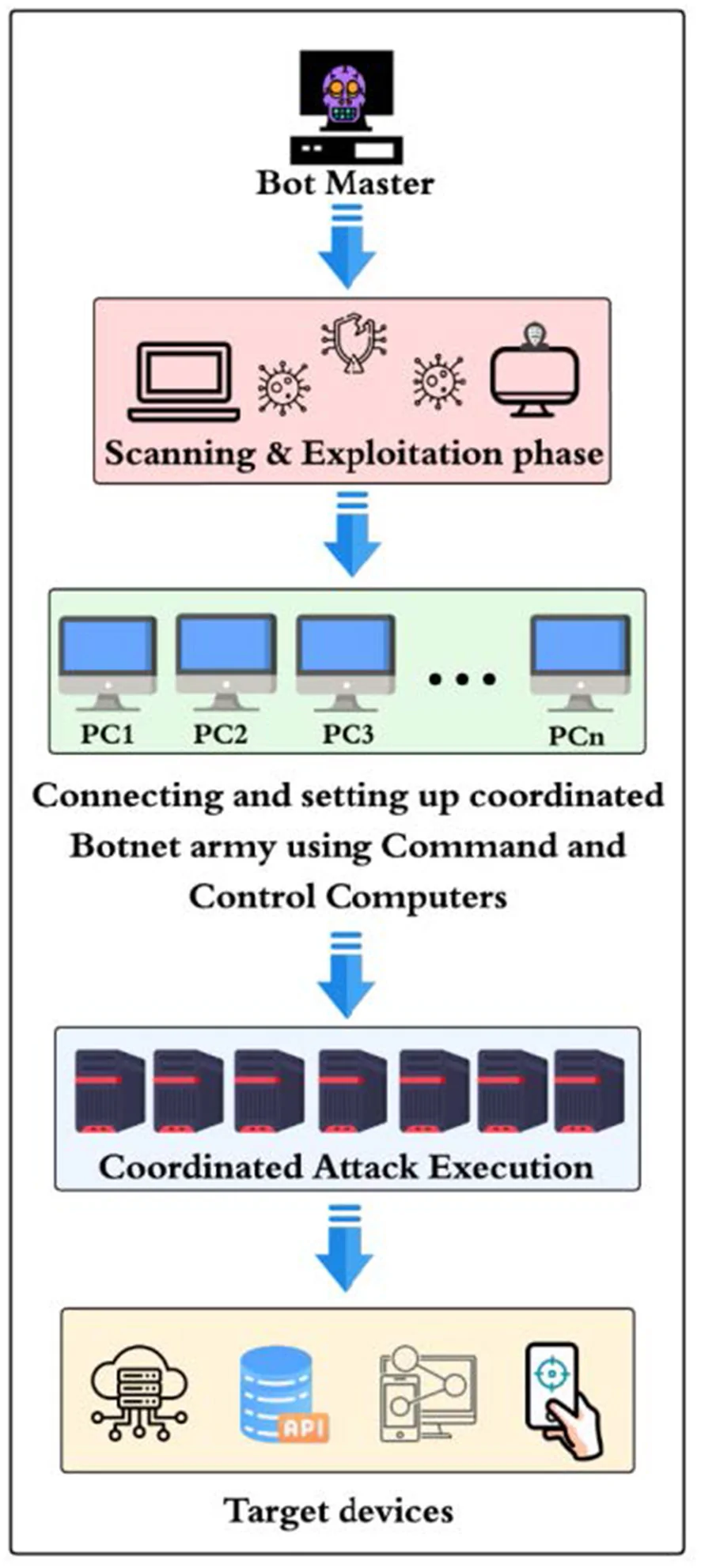
Phases of botnet lifecycle with emphasis on attack evolution.

The Internet of Things (IoT) comprises a network of devices that connect and share data, which can be useful for meaningful tasks. These tasks include smart healthcare services, smart homes, and Industrial IoT (IIoT). Advancements in areas such as 5G networks also increased the widespread use of IoT technologies (Ahmed et al., 2024). Recent IoT analytics statistics show a 14% growth in IoT-connected devices, bringing the total to 21.1 billion devices worldwide. An IoT network has a heterogeneous structure due to the different types of devices that connect to the network. This heterogeneous structure can be unstable and thus increase the ease for attackers to gain unauthorized access to the network (Zahid et al., 2022). Among these attacks, the most destructive ones come from botnet attacks. A botnet attack, as shown in Figure 1, is a cyber-attack where bots—a network of computers that are infected and controlled by a botmaster who carries out tasks such as distributed denial of service (DDoS), spam, and phishing emails (Nazir et al., 2023), coordinate together to form an army of malicious nodes that act together to bring out massive destruction to target devices. The devices that got infected by this process are called zombies. These zombie devices are then controlled by the botmaster, and the owner of the device will not be aware that the attack is taking place. Well-known botnets include Mirai, Hajime (Anh and Nakamura, 2024), IoT Reaper, and Gafgyt (Siddamsetti and Srivenkatesh, 2022). These attacks are fatal to IoT network systems, and given the massive destruction they can cause, there is a need for an excellent botnet detection system to overcome the security issues faced by the systems that are vulnerable to the attacks.

Modern botnets and zero-day attacks need modern solutions for effective detection. Focusing on lightweight solutions can lead to giving less importance to specific features, which may actually add to the efficiency of detection; for example, (Wei et al., 2023) used only packet length features and did not use packet header or content information, which can lead to missing much important information. Scalability is an issue in graph-based methods (Alharbi and Alsubhi, 2021), as in some cases, new graphs have to be created every time a new IP address is dealt with. Signature-based algorithms (Nasir et al., 2023) fail at detecting new or zero-day attacks since they do not have signatures for them. Rule-based intrusion detection systems (IDS) (Ayo et al., 2023) also fail to detect zero-day attacks since they use rules or behaviors that are based on previously known botnets, which will not be applicable to a new and unknown botnet during the detection process. Many machine learning (ML) (Almuqren et al., 2023; Kalakoti et al., 2022; Ibrahim et al., 2021) and deep learning (DL) (Al-Shurbaji et al., 2025; Nadeem et al., 2023; Shahhosseini et al., 2022) methods have also been used to detect botnets, but they still face issues such as data imbalance, lower accuracy in detecting unknown botnet attacks, and high false positive rates. Therefore, there is a need for an approach where unknown attacks can be better identified accurately and effectively and also reduce the issues caused by data imbalance.

Use of classifiers such as support vector machine (SVM) (Masoudi-Sobhanzadeh and Emami-Moghaddam, 2022) has to be double-checked for optimal performance regarding the dataset and type of attacks, since the whole feature selection process can become faulty when the right classifier is not chosen. Even though packet-based intrusion detection reduces the delay in detection (Alani, 2022), flow-based detection gives a broader and detailed view of the activities happening in the network. Another issue of data imbalance occurs with studies that use datasets such as N-BaIoT, where the number of attack samples is much less compared to benign or normal samples. One study (Habibi et al., 2023) used modeling of tabular data using conditional tabular generative adversarial network (CTGAN) and machine learning to reduce the data imbalance problem and thus improve IoT botnet detection, whereas the tabular data being changed can actually be complex and can also cause data structure distortion. In real-time botnet detection models (Velasco-Mata et al., 2023), speed is given more importance compared to accuracy, while efficient botnet detection may need more importance to be given.

This study proposes a deep learning model for IoT botnet detection which uses WGAN for synthetic data generation which will mitigate the data imbalance problem in the dataset. An enhanced prototypical network with attention mechanism, a dual-mode neural architecture, is then operated in two distinct configurations: standard classification mode for standard supervised learning with full dataset and few-shot learning mode for episodic learning with limited labeled examples. The few-shot mode will train the neural network for detection capacity even with a few shots of training samples. The dataset that has been used for this study is the CIC IoT dataset 2023. Few-shot learning will help with detecting new unseen attacks since they have the ability to learn from very few samples, and along with synthetic data from generative adversarial network (GAN), they can perform really well with detecting unknown attacks.

The quick spread of Internet of Things (IoT) devices and botnets, especially MIRAI variants, have become one of the biggest dangers to network security. These botnets cause significant operational and financial harm by taking advantage of weak IoT devices to initiate distributed denial-of-service (DDoS) attacks, credential theft, and other malevolent actions. Botnet detection using traditional machine learning techniques depends on supervised learning using sizable, well-balanced datasets. However, there are three main obstacles in the real-world operational environment that make traditional methods useless: extreme class imbalance, dynamic and evolving botnet behavior, and lack of labeled data for novel attacks.

The main objectives of this study include the following:

1. Develop an adaptive botnet detection framework that can learn from evolving network traffic without catastrophic forgetting of previously seen botnet patterns.
2. Create a single model that can detect botnets in both few-shot scenarios and normal scenarios with ample data.
3. Generate realistic synthetic data to address the extreme class imbalance using WGAN-GP.
4. Detect concept drift using multi-test statistical approach (KS test, Wasserstein distance, and class-prior shift) and execute automatic model update when drift is identified.
5. Verify performance in real-world scenarios with significant noise and class overlap.
6. Provide a thorough assessment utilizing security-specific metrics against state-of-the-art baselines.

This study makes the novel contribution of integrating SSL-pretraining, few-shot learning, continual learning, and drift detection and their adaptation to the botnet detection domain, which together provide capabilities not previously demonstrated in the literature. Previous studies treat these methods in isolation, making this study the first to jointly address label efficiency, adaptation to new attack families, forgetting prevention, and real-time drift monitoring. SSL-pretraining is applied to unlabelled packet-flow data, learning transferable representations that significantly improve few-shot performance. EWC stabilizes continual adaptation, and drift detection decides when to adapt, ensuring the system remains robust under evolving threats.

The remainder of this study is organized as follows: Section 2 presents related work on IoT botnet detection. Section 3 discusses the proposed methodology of the work, and Section 4 describes the experimental setup and results. Section 5 gives the conclusion and future directions of the study.

## 2 Background and related works

Several machine learning and deep learning techniques were used to solve the problem of the inefficiency in botnet detection and the detection of zero-day attacks. Data imbalance issues in benchmark datasets have led to poor generalizability and efficiency of botnet detection models. Even though they achieve good accuracy, data imbalance will lead to poor detection of attacks that come under minority samples. This section discusses various methods that were used for botnet detection, with insights into the methods used, the dataset, and their fallbacks.

### 2.1 Machine learning based methods

Saleh et al. (2026) introduced a federated learning technique to maintain detection performance while keeping up with privacy concerns. This framework works on a horizontal federated learning architecture which uses an average aggregation (FedAvg) algorithm and random client selection while learning. In (Srinivasan and Deepalakshmi, 2023), proposed a system for detecting botnets which uses the ensemble classifier with stacking process (ECASP) that can select optimal features to feed into the machine learning structures thereby improving the detection performance. Another study focused on the time-series-based feature extraction called network intra-flow parameter time series analysis which was done by (Jovanović and Vuletić, 2025). The method then uses XGBoost and LightGBM gradient boosting models for classification. According to (Taher et al., 2023), a novel feature selection approach was introduced which selects the most important features through clustering that rank features and apply Grasshopper Algorithm (GOA) to minimize top-ranked features. Table 1 shows a summary of the works done using machine learning for botnet detection in recent years.

**Table 1:** Various machine learning approaches that have evolved over time in botnet detection.

References	Method	Dataset	Results	Drawbacks
Saleh et al. (2026)	Federated learning (FL) techniques	N-BaIoT	Accuracy of 87.5%, precision of 92%, and F1-score of 0.89.	Communication overhead, overfitting.
Srinivasan and Deepalakshmi (2023)	Ensemble classification with stacking process (ECASP)	Cyber Clean Center (CCC) dataset	Accuracy of 94.08%, Sensitivity of 86.5%, Specificity of 85.68%, and an F-measure score of 78.24%.	Flow-based detector limitations
Jovanović and Vuletić (2025)	Network intra-flow parameter time series analysis, machine learning classification	ETF-IoTB dataset	Accuracy of 97.06% and F1-score of 0.9041.	Inherent dataset imbalance, overfitting.
Taher et al. (2023)	Grasshopper Algorithm (GOA), Harris Hawks Algorithm	N-BaIoT dataset	Accuracy of 98.07%, precision of 97.04%, recall of 98.73%, and F1-score of 97.87%.	Limited model generalization, struggle with high traffic volumes.

### 2.2 Deep learning and hybrid methods

The use of deep learning models increased the capability of botnet detection models to a great extent, with their tability to automate tasks such as feature learning. Sakthipriya et al. (2024) proposed a framework that effectively addresses the challenges of botnet attack detection that integrates a conditional adversarial autoencoder (CAAE) for feature extraction and a dilated and cascaded recurrent neural network (DC-RNN) for attack classification. Studies also showed good results with hybrid frameworks, which were shown in the work done by Bindu et al. (2025) demonstrating combination of recurrent neural networks (RNNs) and extreme learning machines (ELMs). One of the current challenges being faced by the security domain is finding zero-day attacks. Lohani and Sangal (2025) in their work explain an adversarial resilient asynchronous federated learning framework for zero-day IoT botnet detection which uses multi-layer perceptron model for detection and showed high classification performance on both known and zero-day attacks. Another study that attempts to solve zero-day botnet attacks in Android devices was done by (Seraj et al., 2025), where a novel method using multi-layer perceptron (MLP) and a novel dataset was also created by the authors. In spite of these developments, existing deep learning methods still undergo challenges such as difficulty in coping with the rapid evolution of botnet attack styles, reliance on labeled data that affects zero-day attack detection, severe class imbalances in datasets, and data dependence. A summary of the deep learning studies in recent times is portrayed in Table 2.

**Table 2:** Overview of recent deep learning methods for botnet detection.

References	Method	Dataset	Results	Drawbacks
Sakthipriya et al. (2024)	Cascaded deep learning, conditional adversarial autoencoder (CAAE)	N-BaIoT	96%, 98%, 97%, and 96% accuracy, precision, F1-score, and recall	Scalability, RNN limitations—vanishing and exploding gradient problems
Bindu et al. (2025)	RNN, extreme learning machines (ELM), American Zebra Optimization Algorithm (AZOA)	N-BaIoT	Accuracy of 96.60%	False positives in anomaly-based and signature-based methods
Lohani and Sangal (2025)	MLP with ReLU activation function, federated learning	N-BaIoT	Peak F1-score of 99.85%	Identically distributed data landscape, varying adversarial resilience across devices
Seraj et al. (2025)	MLP with sigmoid activation function	Android Botnet dataset (created by authors)	Accuracy of 98.5%, Precision of 98.3%, and recall of 98.8%	Reliance on static analysis

Few-shot learning (Xu et al., 2020; Lu et al., 2023) is a very adaptive way of enhancing the detection of zero-day attacks since the algorithm has very strong learning skills where patterns are learned even with very few samples, but still the efficiency of few-shot learning comparing to a complete dataset-based training has to be proved. Prototypical networks form the base for few-shot learning algorithm where they make a prototype for each class using the given few numbers of samples (Malik et al., 2025; Miao et al., 2023). Study (Xu et al., 2025) uses datasets such as ISCX-IDS2012, CICIDS2017, and CICIDS2018 in an adaptive way where a few-shot learning scenario is created since all datasets have more than enough samples and do not come under few-shot criteria. A unified approach of few-shot learning and prototypical networks would pave the way for an excellent detection system since many of the real-world zero-day attacks would have very few samples for training data.

Continual learning has emerged as a valuable method for enabling machine learning models to learn from sequentially arriving data and, at the same time, preserving prior knowledge. One of the studies (Ngoc, 2026) shows effective mitigation of catastrophic forgetting in non-stationary data environments by using a continual learning framework for GANs. Self-supervised learning (SSL) has gained increasing attention in botnet detection due to the limited availability of labeled attack data and the continuously evolving nature of cyber threats. (Fan et al., 2026) proposed Anomal-EFD, a self-supervised anomaly detection framework for dynamic IoT networks based on graph neural networks. The model combines a random sliding window mechanism to capture temporal network evolution, a multi-head self-attention module to aggregate neighborhood information, and contrastive self-supervised learning to learn discriminative node representations without requiring extensive labeled data. Transformer learning employs self-attention mechanisms to learn global feature representations from input data. Its parallel processing capability and ability to model long-range dependencies make it well suitable for network intrusion detection, particularly in complex and high-dimensional traffic environments. A recent study (Zhao et al., 2025) uses a transformer-based intrusion detection framework named DTF-VPP, for virtual power plant networks. It combines principal component analysis (PCA) for feature filtering with a transformer encoder employing multi-head self-attention to capture global feature dependencies. (Mokadem et al., 2026) proposed an adaptive network-oriented cybersecurity framework that integrates an AI coach to support organizational learning and risk management in cybersecurity teams. The framework uses adaptive network modeling with first- and second-order learning mechanisms to simulate cognitive adaptation, shared mental models, and AI-assisted decision support under different cybersecurity scenarios.

### 2.3 Research gaps and motivation

Even though a very large amount of research has been conducted on IoT botnet detection, most of the methods used still face problems, such as data imbalance, where the dataset would have very few instances of a particular class, which would lead to poor classification. Most studies also depend on public datasets, which are already imbalanced, having more of either attack instances or benign instances. Some datasets are taken in environment settings where attacks continuously happen, and this dataset will have an imbalance toward more instances of attacks than benign instances and vice versa. To address these gaps, this study proposes a novel deep learning framework consisting of WGAN-GP along with few-shot learning and enhanced prototypical network with attention mechanism, where the GAN structure will improve the data imbalance of already existing public datasets by generating more samples of the minority instance of data, and the power of the few-shot learning architecture would make it simple and even to learn from those classes which have very few numbers of instances, together making it an excellent framework for solving the problems in IoT botnet detection.

## 3 Proposed methodology

Traditional botnet detection systems work on the assumption that the network traffic patterns remain static over time. But in real world, botnets evolve rapidly over time which will impact the level of detection. Botnets evolve through concept drift, feature drift, and class imbalance evolution. Concept drift occurs when the relationship between the input features and the attack label changes over time, that is, the attack behavior changes. For example, Mirai botnet variants normally performed Transmission Control Protocol Synchronize (TCP SYN) flooding, Hypertext Transfer Protocol (HTTP) flooding, and simple scanning behavior. But new Mirai variants may introduce encrypted communication, randomized packet intervals, different command and control mechanisms, and adaptive attack timing, which is very different from the prior tactics of attack. So, with traditional botnet detection systems, if a botnet attack behavior looks like high SYN rate, then the model may flag it as attack. But in real scenario, the botnet may take a new strategy, that is moderate SYN and encrypted payload which is actually an attack, but which may not be identifiable by traditional botnet detection systems and the decision boundary becomes outdated.

Feature drift is the statistical change in network data over time. For example, while training, a sample with packet size 300 bytes and 2-s flow duration may have been marked as benign, but after deployment an actual normal sample may have 700 bytes of packet size and flow duration of 5 s, which will be falsely classified as an attack. Traditional systems consider the network data to be not changing and to be static which results in increased false positives, model instability, and reduced generalization. Another problem faced is class imbalance evolution, where the dataset has different proportions of classes, say 70% attack data and only 30% benign data, or vice versa. The problem here is that models get biased toward the dominant class. If benign classes are more, the model may even classify the attack as a benign label, again here creating false alarms, poor recall for minority class, and false positives.

**Figure 2:**
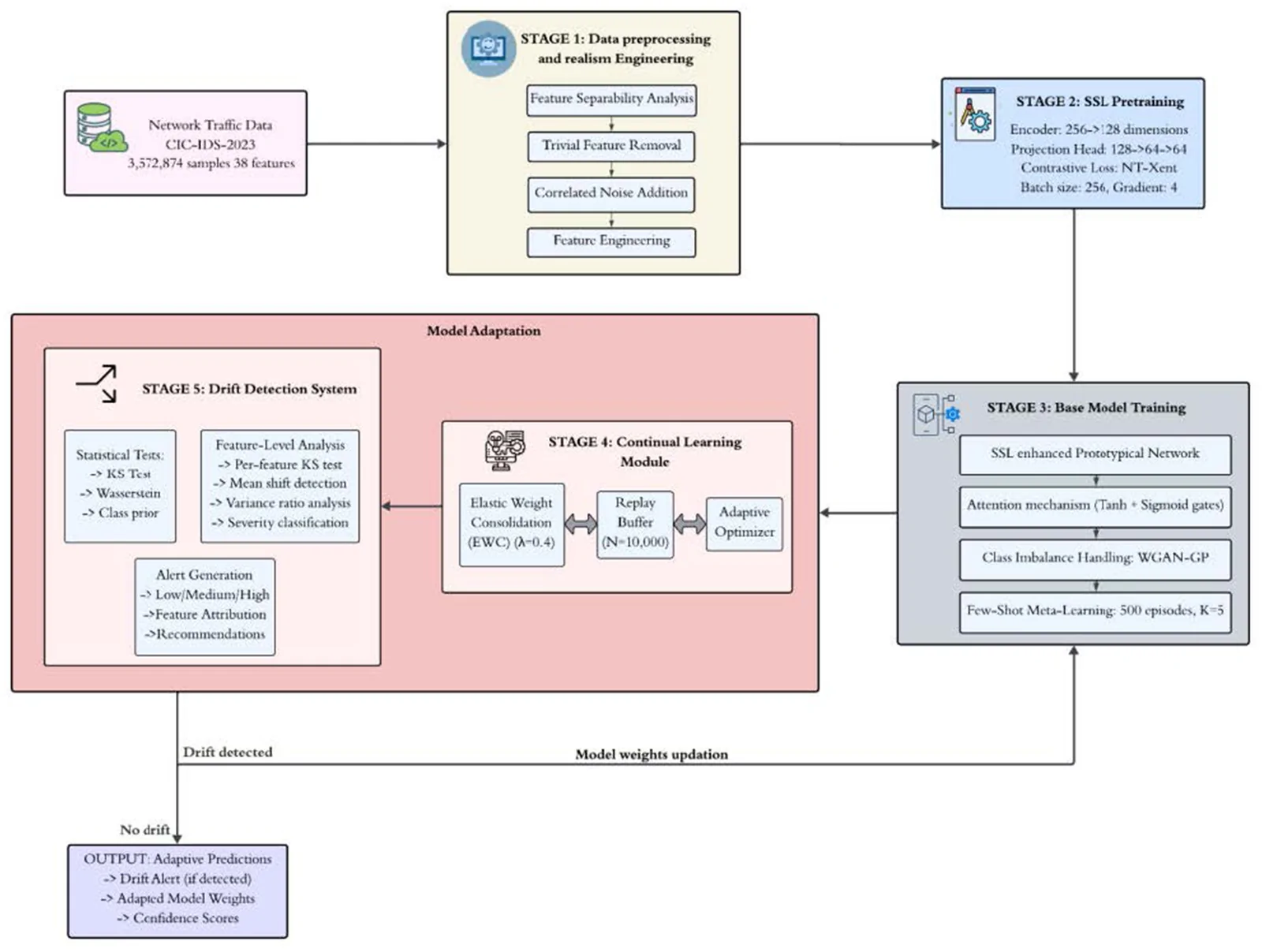
Overall architecture of the SSL-enhanced prototypical network with few-shot learning capability.

To mitigate the problem of unknown or zero-day attacks in the existing literature, the proposed model in this research uses a novel integration of continual learning with drift detection for botnet detection. Feature-level drift analysis with severity-based alerting system is also implemented for efficient detection. Elastic weight consolidation (EWC) is adapted to prototypical network to eradicate catastrophic forgetting. Multi-test statistical drift detection is done using KS test, Wasserstein distance, and class-prior shift. Adaptive few-shot learning is incorporated in this research which helps in botnet detection where scenarios come when only few samples of an attack are available for training. The overall architecture of the proposed framework is depicted in Figure 2.

### 3.1 Data processing and realism engineering

Models often fail in real-world deployment because of the lack of realism in their data due to perfect separation whereas real network traffic will have overlaps between classes and has missing values and variations. Normally botnets in the dataset are taken to be stationary whereas actually they keep on evolving. The solution is to make the dataset realistically challenging while maintaining the ground truth.

#### 3.1.1 Feature separability analysis

Feature separability analysis measures how well each feature can distinguish classes, using Equation 1. For each feature *f*_i_ in the dataset, compute:


$$\begin{array}{l} S\left(f_{i}\right)=2\times |AUC\left(f_{i}\right)-0.5| \end{array}$$ (1)

where AUC (*f*_i_) is the area under the receiver operating characteristic (ROC) curve when using only feature f_i_ to classify, and *S*(*f*_*i*_)∈[0, 1] is the separability score.

From the dataset, features such as Number, Header_length, Protocol Type, and HTTPS were trivial features and inter-arrival time (IAT) and Bluetooth low energy (BLE) rate were highly discriminative features, whereas Min and AVG were moderately useful features. Table 3 illustrates the separability score, interpretation, and the action taken for each score.

**Table 3:** Interpretations of separability scores showing the criteria for feature elimination.

Separability score	Interpretation	Action
*S*>0.9	Trivial feature—almost perfect separation	Remove or add overlap
0.7 < *S* ≤ 0.9	Highly discriminative	Add noise
0.5 < *S* ≤ 0.7	Moderately useful—realistic	Keep the feature
*S* ≤ 0.5	Poor feature—not useful	Consider removal

#### 3.1.2 Trivial features removal

For removal of the features that are real, a threshold value is set, θ as 0.9. The features that would be kept are the set of all features (Fall) excluding the trivial features (Ftrivial). The trivial features are selected using Equation 2, and the features that are retained are represented using Equation 3. By doing this, features such as Header_Length, Number, RCP, Tot sum, Protocol Type, ack_flag_number, HTTPS, and ack_count are removed, and the remaining 22 features are kept. Table 4 gives an insight into various trivial features and their justification to be taken as trivial.


$$\begin{array}{l} F_{trivial}=\left\{f_{i}|S\left(f_{i}\right)>\theta \right\} \end{array}$$ (2)

$$\begin{array}{l} F_{kept}=\;F_{all}\backslash F_{trival} \end{array}$$ (3)

**Table 4:** List of trivial features with justification for triviality of each feature.

Removed feature	Reason for triviality	Real-world issue
Header_Length	Fixed headers	Modern botnets randomize headers
Number	Perfect packet count separation	Varied traffic volume
TCP	Protocol exclusivity	Botnets use multiple protocols
Tot sum	Size-based separation	Encryption hides sizes
Protocol Type	Protocol-based detection	Botnets mimic normal traffic
ack_flag_number	Uniqueness of flag patterns	Flags can be spoofed
HTTPS	Port-based detection	Blends encrypted traffic
Ack_count	ACK pattern uniqueness	Randomized patterns of botnets

#### 3.1.3 Correlated noise addition

**Figure 3:**
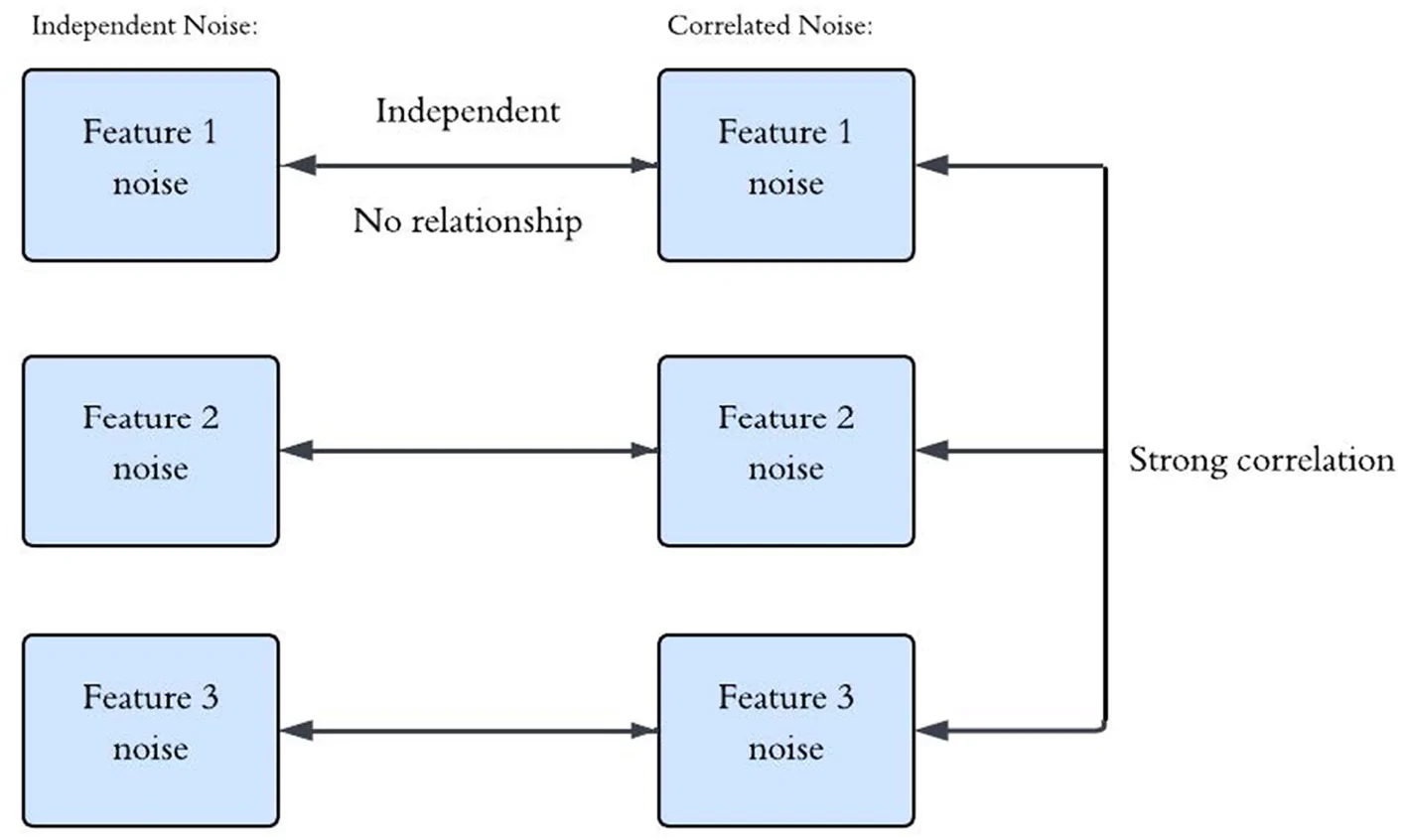
Representation of correlated noise addition and interconnection between features.

Correlated noise addition is the process of adding interdependent noise to features rather than adding noise to each feature, as shown in Figure 3. This will create a real-world scenario where network measurements are affected by similar changes in the traffic by botnets. Instead of adding independent noise,


$$\begin{array}{l} \epsilon_{i}\sim N\left(0,\;\sigma_{i}^{2}\right) \end{array}$$ (4)

Correlated noise is added to all the features:


$$\begin{array}{l} \epsilon \sim N\;\left(0,\;\Sigma \right) \end{array}$$ (5)

Equation 4 represents how independent noise is added to features, and Equation 5 shows correlated noise addition. where **Σ** is a covariance matrix that measures relationship between features.

#### 3.1.4 Feature engineering

Feature engineering is the process of creating new informative features from the existing data. Here, ratio features and correlated patterns are created. A single feature may not always give the deep interconnections that exist between data, and therefore, the creation of correlated features will aid in better understanding of the data.

Ratio features are created by dividing one existing feature by another. They capture proportional relationships that cannot be identified by a single feature. To capture proportional relationships that are stable across network environments, pairwise ratios of the first four original features are computed. For any two features a and b, ratio feature can be created using Equation 6:


$$\begin{array}{l} r_{a.b}=\;\frac{a+\epsilon }{b+\epsilon } \end{array}$$ (6)

The ratio is then z-scored (mean-centered, scaled by standard deviation) to standardize the magnitude. For training and validation sets, Gaussian noise is added with standard deviation to prevent overfitting and simulate realistic variability. No noise is added to the test set, ensuring a fair evaluation. These ratio features are appended to the original feature set. A Random Forest classifier (100 trees, max depth 10, balanced class weights) is trained on up to 200,000 samples from the training set. Feature importance is measured by the mean decrease in impurity (Gini importance). The top 30 most important features are selected to reduce dimensionality and remove redundant/correlated features. To prevent overly optimistic performance and to mimic real-world botnet detection where no single feature perfectly separates benign from malicious traffic, features are removed that are too discriminative. For each feature, its single-variable ROC-AUC is computed against the binary label. This step reduced the feature set from 30 to 22. Finally, all features are normalized to the [0, 1] range using a Min-max scaler fitted exclusively on the training data. The same scaler is applied to the validation and test sets to prevent data leakage.

The realism engineering method will create a harder, more realistic benchmark but is not intended to replace the original dataset. To ensure comparability, a reference model is also trained on the original, unmodified training data and evaluated on the same original test set.

### 3.2 Self-supervised pretraining

Self-supervised pretraining is a procedure where a model learns useful representations from unlabelled data by creating its own supervision signals from the data itself. This is needed since unknown attacks do not come with a label, and new variants emerge constantly, and thus, this technique will make the model resilient to unknown and modified attacks also. Self-supervised learning (SSL) includes the network traffic, data augmentation module, encoder network, projection head, and contrastive loss which is normalized temperature-scaled cross entropy (NT Xent) loss. SSL stores each sample's network features as feature vectors (22 features per sample). So initially it is in the form represented in Equation 7:


$$\begin{array}{l} x_{i}\;\epsilon \;{\mathbb{R}}^{22} \end{array}$$ (7)

Then, each sample is augmented into two different versions, which are called two views of the same sample. Each view will be slightly different from the original sample of the network flow in different ways, which would not change the meaning of the original sample. Each sample *x*_i_ is augmented as shown in Equation 8:


$$\begin{array}{l} x_{i}\to v_{i}^{1},\;v_{i}^{2} \end{array}$$ (8)

The encoder network converts the input representation to a compact form to retain only the most important information and get rid of any noise or redundant details. The encoder consists of two fully connected layers. The first layer projects the input from 22 features to 256 dimensions, followed by batch normalization, a Rectified Linear Unit (ReLU) activation and dropout with a rate of 0.4. The second layer compresses the 256 features to 128 more abstract features, again followed by batch normalization, ReLU activation, and dropout with a rate of 0.3. This architecture helps the model learn meaningful patterns more efficiently. The encoder transforms the augmented sample to learned representation using Equation 9:


$$\begin{array}{l} v_{i}\to h_{i} \end{array}$$ (9)

where *v*_*i*_ is the augmented sample, and *h*_*i*_ is the learned representation or embedding. The embedding narrows down the important network traffic patterns. After the encoder, the representation is passed on to the projection head. The projection head increases the quality of the representation and also preserves the original encoded representation for later use. There will not be any changes in dimensions. The projection head is represented in Equation 10.


$$\begin{array}{l} h_{i}\to z_{i} \end{array}$$ (10)

Where, *h*_*i*_ is the encoder output, and *z*_*i*_ is the projection vector used for contrastive learning. The next process in the pipeline is contrastive loss using NT Xent loss, where the goal is to pull similar samples together and push dissimilar samples apart. This will make the embeddings of similar network traffic flow together and dissimilar ones away. As the output, the encoder learns meaningful representations and these representations can be used for botnet detection. The projection head is removed, and the encoder output can be used as features for classifier.

### 3.3 SSL-enhanced prototypical network with attention

#### 3.3.1 SSL-enhanced encoder

**Figure 4:**
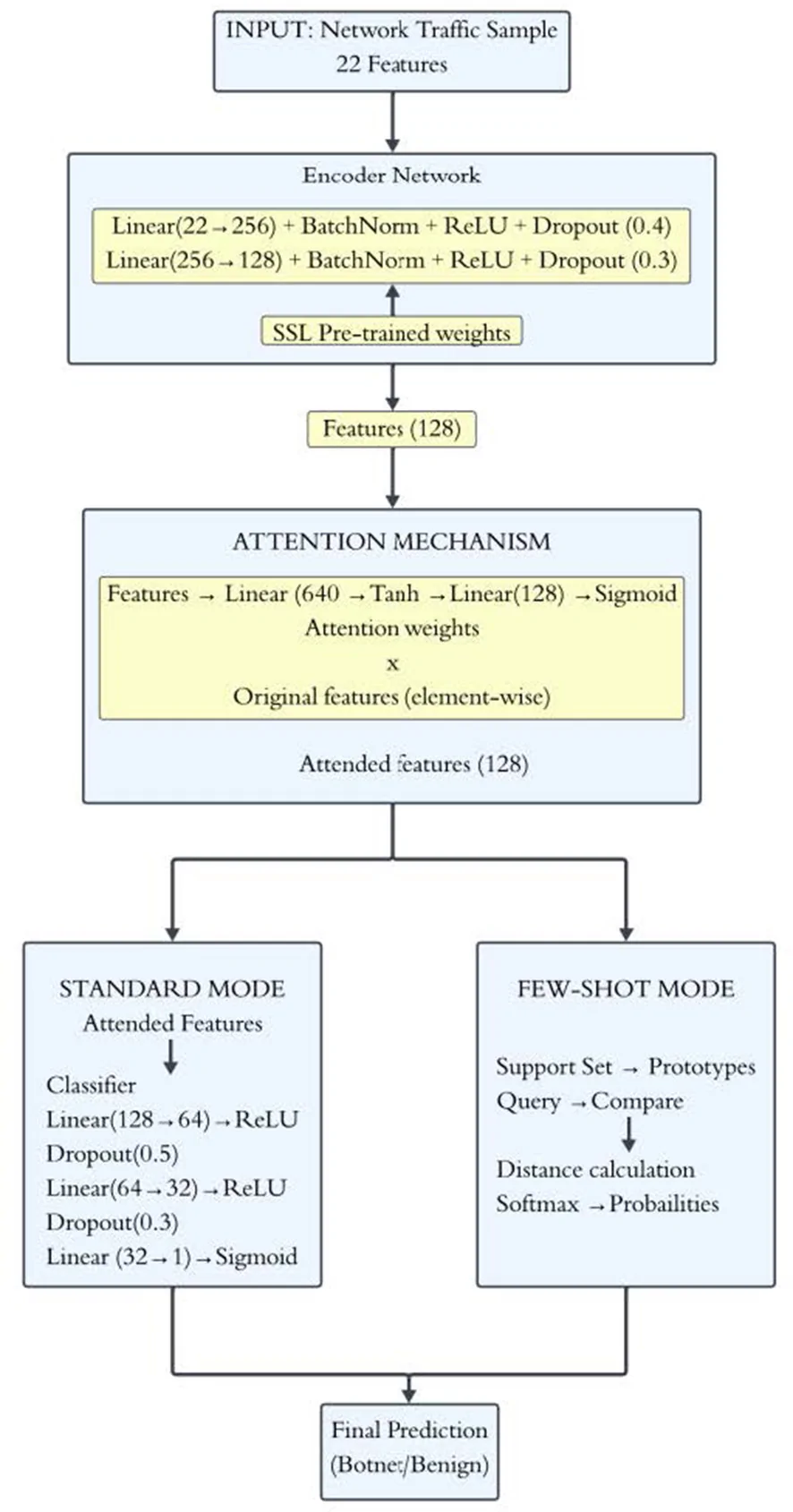
Workflow of the base model: SSL-enhanced prototypical network with attention mechanism.

The encoder is trained from the weights learned during the SSL-pretraining process in the previous module. Without SSL-pretraining, the model should have needed to start from random weights whereas here we can use weights that have already understood the network traffic patterns. With SSL-pretraining, we need only a few labels for prediction which will be well suited for few-shot learning and efficiently reduce the training time by reducing the number of epochs also. The detailed architecture of the network is shown in Figure 4.

#### 3.3.2 Attention mechanism

After training, the attention weights indicate which features matter the most for detection. The encoder produces a feature representation for each input sample. Rather than treating each feature equally, the attention mechanism computes importance of weights which would produce attention weighted features. The weighted features are passed through a fully connected layer with 64 output units, and the bias vector provides each of the 64 neurons with an independent bias which gets activated at different thresholds. For input features **f**∈ℝ^128^, attention and attended features are calculated by using Equations 11 and 12 respectively.


$$\begin{array}{lll} \text{a} & = & \sigma \left({\text{W}}_{2}\tanh\left({\text{W}}_{1}\text{f}+{\text{b}}_{1}\right)+{\text{b}}_{2}\right) \end{array}$$ (11)

$$\begin{array}{lll} {\text{f}}_{attended} & = & \text{f}⊙\text{a} \end{array}$$ (12)

where ${\text{W}}_{1}\in \;{\mathbb{R}}^{64\;\times \;128}$, ${\text{b}}_{1}\in {\mathbb{R}}^{64}$, **W**_1_ being the weight matrix of the first linear layer, and **b**_1_ the bias vector. In the second linear layer, the dimensions are expanded back to the original feature size ${\text{W}}_{2}\in \;{\mathbb{R}}^{\left\{128\times \;64\right\}}\;,{\text{b}}_{2}\in \;{\mathbb{R}}^{128}$, and tanh introduces non-linearity with σ that maps the output to [0, 1] as attention weights and ⊙ is element wise multiplication.

##### 3.3.2.1 Dual mode operation

The model operates in standard and few-shot mode, providing detection in both scenarios according to the demand of the situation of the availability of the data for training. The classification module receives the attention-weighted feature representations (128 dimensions) and processes them through a series of fully connected layers. The first hidden layer reduces the dimensionality from 128 to 64 neurons, applying ReLU activation followed by a dropout regularization to 32 neurons, again using ReLU activation and dropout with probability 0.3. The output layer consists of a single neuron with sigmoid activation, producing a probability score in the range [0, 1] which indicates the likelihood of the input being botnet traffic.

In few-shot classification, Euclidean distance is measured to find how closer a query is to a prototype representation since prototypical networks work on the principle that classes should cluster around their prototypes in the feature space. The closer the query is to a prototype, the more are the chances that it belongs to that class. During training, the model alternates between the two modes, and while testing, either one of the modes can be selected for operation. SSL-pretraining gives good support for predictions since the encoder in the framework already understands the network traffic well so that it can understand a normal or attack pattern even before it sees a label.

##### 3.3.2.2 Prototypical network formulation

Prototypical network is a few-shot learning architecture that classifies examples by calculating the distance between prototype representations and classes. It learns an embedding space where examples from the same class cluster together around a prototype instead of learning a complex decision boundary. The process starts with extracting attended features; for any input sample *x*, it is first passed through the encoder and attention mechanism. Equation 13 explains the extraction of attended features.


$$\begin{array}{l} f_{attended}\left(x\right)=Attention\left(Encoder\left(x\right)\right)\in {\mathbb{R}}^{128} \end{array}$$ (13)

This is a 128-dimensional vector that represents the sample in a space where similar samples are close together and different classes are far apart. The next step computes prototypes for each class, where a support set *S* will have *K* examples per class as described in Equation 14.


$$\begin{array}{l} S=\left\{\left(x_{1},y_{1}\right),\;\left(x_{2},y_{2}\right),\ldots ,\;\left(x_{n},\;y_{n}\right)\right\} \end{array}$$ (14)

where *x*_*i*_ is input sample or feature vector, *y*_*i*_ is the class label, and *n* is the total number of support samples. It is represented as *n*_*way* and *k*_*shot*, which means number of classes and number of samples per class, respectively. So, for example if *n*_*way*_ = 2 and *k*_*shot*_ = 5, there will be a total of 2 × 5 = 10 labeled samples in the support set. For each class c, samples are collected which belongs to that class, as represented in Equation 15.


$$\begin{array}{l} S_{c}=\left(x,y\right)\in S\;:y=c \end{array}$$ (15)

Here, *S*_*c*_ will contain all the elements that belongs to the class c. Then, the prototype for each class is computed as the mean of feature vectors belonging to that class, as depicted in Equation 16. Here, for botnet classification, prototype will be calculated for benign and botnet classes.


$$\begin{array}{l} p_{c}=\;\frac{1}{\left|S_{c}\right|}\;\underset{\left(x,y\right)\;\in \;S_{c}}{\sum }f_{attended}\left(x\right) \end{array}$$ (16)

where |*S*_*c*_| denotes the number of samples in class c. A query sample is compared with each class prototype to decide its class. Let *x*_*q*_ be the query, a new IoT network flow. The model has to decide whether it is benign or a botnet traffic. First, the query is passed through the encoder and attention module, which produces a of 128-dimensional feature vector *f*_*attended*_(*x*_*q*_). Then, the distance is calculated between the query feature and each of the prototypes using the Euclidean distance formula, as shown in Equation 17.


$$\begin{array}{l} d\left(f_{attended}\left(x_{q}\right),p_{c}\right)=\;\overset{128}{\underset{i=1}{\sum }}{\left(f_{attended}{\left(x_{q}\right)}_{i}-p_{c,i}\right)}^{2} \end{array}$$ (17)

where *f*_*attended*_(_*x*_*q*_)*i*_ is the *i*^*th*^ feature of the query sample, *p*_*c, i*_ is the *i*^*th*^ feature of the class prototype, and *d* is the distance. For final prediction, the distances have to be converted to probabilities that sum to 1 using the Softmax function, using Equation 18.


$$P(y=c\;|\;x_{q})=\frac{exp\left(-d\left(f_{attended}\left(x_{q}\right),p_{c}\right)\right)}{\sum_{c^{\prime }=1}^{C}exp\left(-d\left(f_{attended}\left(x_{q}\right),p_{c^{\prime }}\right)\right)}$$ (18)

where *x*_*q*_ is the query sample, *p*_*c*_ is the prototype of class c, *d* is the Euclidean distance, and *C* is the number of classes. Since Softmax gives higher probability to larger values, negative of the distance is taken for calculation.

#### 3.3.3 Class imbalance handling with WGAN-GP

When classes are imbalanced, standard machine learning models become biased toward the majority class, whether they are benign or botnet here. In CIC IoT dataset 2023, 29.4% of the data is benign and the remaining 70.6% data belongs to botnet attacks, which makes botnet attacks a majority class here.

##### 3.3.3.1 Wasserstein GAN with gradient penalty

**Figure 5:**
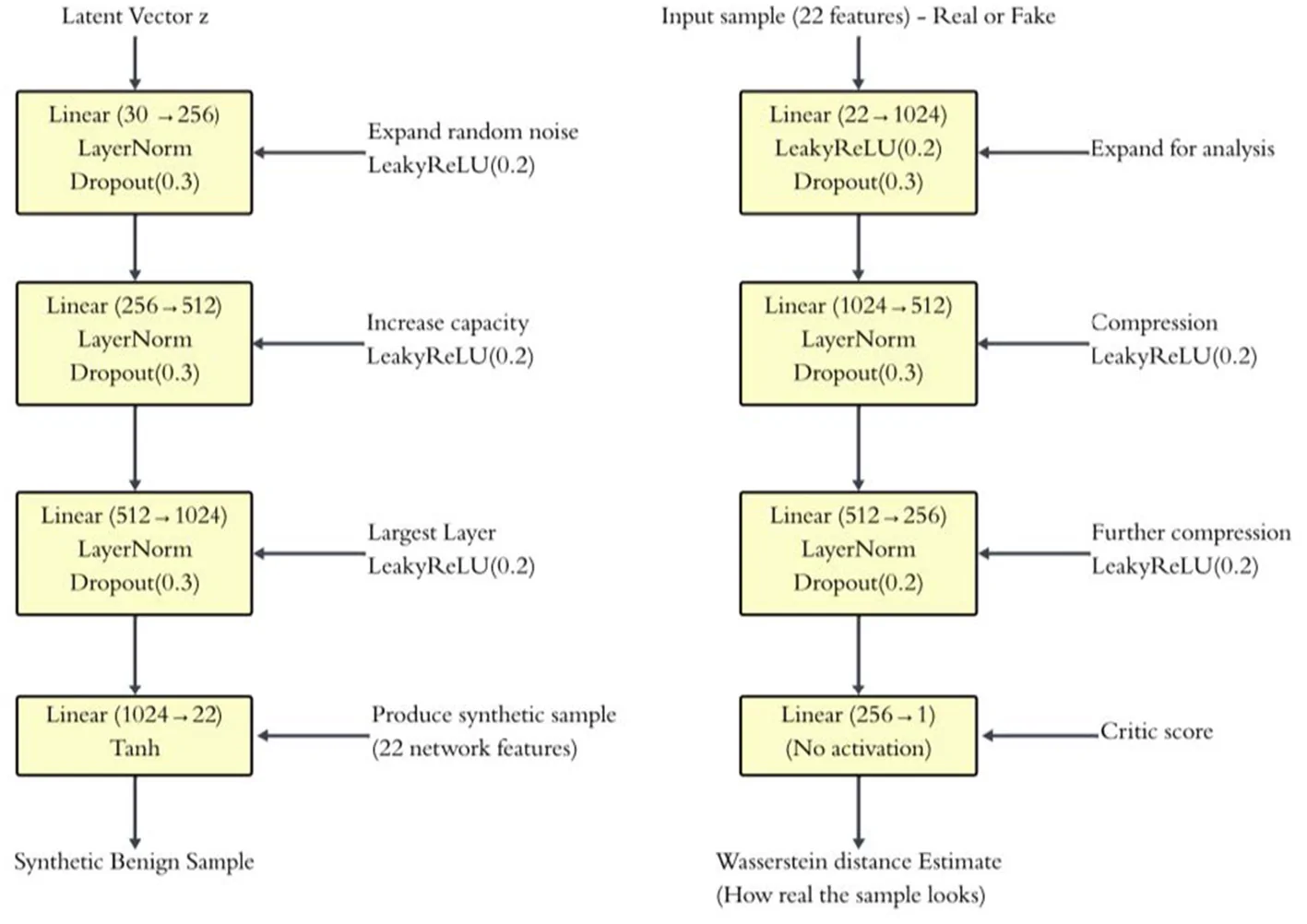
Detailed architecture of generator and discriminator in WGAN-GP framework.

A generative adversarial network consists of two networks, the generator and the discriminator competing toward each other. The generator generates fake samples to fool the discriminator, and the discriminator tries to distinguish between real and fake samples. Different from the standard GAN structures, WGAN-GP calculates Wasserstein distance which gives meaningful gradients, along with gradient penalty. Gradient penalty is used to ensure that the critic's or discriminator's output is not changing with small changes in input. For that, the discriminator should satisfy a condition called 1-Lipschitz constraint. The gradient penalty formula is calculated as in Equation 19:


$$GP=E_{\hat{x}\sim P_{\hat{x}}}\left[{\left(||\nabla_{\hat{x}}D(\hat{x})|{|}_{2}-1\right)}^{2}\right]$$ (19)

where $D\left(\hat{x}\right)$ is discriminator output, $\hat{x}$ is the interpolated sample between real and fake, $P_{\hat{x}}$ is the distribution of interpolated samples, $\nabla_{\hat{x}}D\left(\hat{x}\right)$ is the gradient of critic's output with respect to the input, and *E* is the expectation or average over samples. The gradient penalty enforces the 1-Lipschitz constraint. This will penalize the discriminator if its gradient norm deviates from one. Synthetic samples are generated with the help of WGAN generating 500,000 synthetic samples of benign samples to decrease the imbalance, which were added only to the training set (original training set size: 2,858,298). Additional noise is added to the samples to make them more realistic since GAN generated synthetic samples can sometimes be too perfect. After augmentation, the percentage of benign samples raises to 39.9% and botnet samples decrease to 60.1% giving a better ratio between benign and botnet samples. WGAN augmentation helps in benign and botnet recall and overall accuracy. The detailed architecture is given in Figure 5.

Three balancing strategies were evaluated on the probe dataset using a Random Forest classifier as a proxy—class weights, simple oversampling, and WGAN-GP. While class weights achieved the highest F1-score on the probe, WGAN-GP was chosen for the full pipeline due to its generative modeling, improved generalization, and quality assurance. WGAN-GP learns the underlying data many times more of the minority class, producing synthetic samples that capture the feature distribution rather than simply duplicating existing instances. By generating novel, realistic samples, WGAN-GP helps the model generalize better to unseen attack patterns compared to simple oversampling. The imbalance is intentionally chosen to be reduced to 1.50:1 ratio rather than fully balancing to 1:1 for two reasons—avoiding overfitting and preserving real-world relevance. Excessive synthetic data, especially from a GAN that may not perfectly capture the true distribution, can introduce noise and cause the model to overfit to artificially generated patterns. In practice, botnet traffic is often more prevalent than benign traffic in certain scenarios. Maintaining a slight imbalance reflects operational realities and prevents the model from being overly biased toward benign traffic.

To verify the realism of the WGAN-GP generated samples, three metrics were employed—maximum mean discrepancy (MMD^2^), the 1-Wasserstein distance, and the accuracy of a discriminator trained to distinguish real from synthetic data. MMD^2^ score obtained was 0.0947 indicating moderate similarity. Wasserstein distance scored 0.0974 showing small distributional shifts and discriminator accuracy of 0.9977, showing that the GAN has not fully converged. To compensate, a small noise perturbation was applied to the synthetic samples, which improves their diversity.

#### 3.3.4 Few-shot meta-learning

Few-shot meta-learning is an approach where a model will learn from very few examples. Instead of learning classes, it learns to adjust to new classes with minimal data. The concept of episode-based learning is used, where, instead of learning from individual samples, episodes are created. Each episode creates a few-shot learning scenario, containing a set of support samples and query samples. Support set has samples for learning, and query set has samples that need to be classified. Here, 500 episodes are created with k-shot = 5 and *n*-shot = 2, which means five samples per class in the support set and two classes—botnet and benign.

### 3.4 Continual learning module

Continual learning helps the model to continuously learn from new experiences while preserving the previously learned botnet variants. This kind of learning is necessary for botnet detection because unlike other attacks, botnets continuously change their behavior to fool the detection process and continue their unknown spread of attacks. Traditional botnet detection systems are static; if trained once then they work the same thereafter, so that if a new variant comes they are unable to classify it. Continual learning consists of three integrated components—elastic weight consolidation (EWC), experience replay buffer, and adaptive continual learner.

EWC prevents catastrophic forgetting by remembering or preserving previous weights during the new training. It estimates the parameter importance or the so-called Fisher information. The Fisher information generates a parameter importance map from the model. EWC regularization loss is computed using Equation 20:


$$\begin{array}{l} L_{EWC}=\;\frac{0.4}{2}\underset{i}{\sum }{F_{i}\left(\theta_{i}-\theta_{old,i}\right)}^{2} \end{array}$$ (20)

Experience replay buffer maintains a set of past experiences that can be replayed during adaptation to prevent forgetting. Reservoir sampling method is also used to give equal probabilities to all samples in a buffer eliminating the process of discarding the oldest samples. The buffer is designed to hold 10,000 samples, large enough to maintain diversity and small enough to not cause more computational complexity. Samples are not randomly selected for replay, importance score is used to select which samples are most valuable—samples in minority class, in decision boundaries and from periods of high drift are given more preference. When a new sample arrives in the buffer, it does not randomly replace the existing sample in the buffer but checks the importance score and replaces the sample that has least importance. This ensures that the buffer does not just maintain diversity, but also quality.

Adaptive continual learner integrates EWC, replay buffer, and drift detection into a unified system that decides automatically when to adapt. It is also known as orchestrator, since it coordinates and controls when EWC and replay buffer about when and what to adapt and what to do with the results. It does drift detection, feature-level analysis, replay buffer update, adaptation if needed, and finally Fisher update.

In the experiments, the continual learning and drift detection modules are evaluated in a simulated streaming setting using the test set. The continual learner is initialized with a random subset of the training data (50,000 samples). This establishes the reference distribution (mean, standard deviation, and class prior) used by the drift detector. Test set partitioning: the entire test set is partitioned into sequential windows of fixed size DRIFT_WINDOW_SIZE = 1,000. These windows are taken in the order they appear in the test array, which itself is a random stratified split of the original dataset (not temporally ordered). For each incoming window, statistical divergences are computed between the window's feature distribution and the reference distribution using the Kolmogorov–Smirnov (KS) test and the Wasserstein distance. Additionally, the shift in class priors is monitored. A composite drift score is calculated (weighted combination of these metrics) and compared against a threshold (DRIFT_THRESHOLD = 0.05). If the score exceeds the threshold, a drift event is flagged. When drift is detected, the model undergoes a rapid adaptation using a combination of elastic weight consolidation (EWC) to penalize changes to important parameters, thus mitigating catastrophic forgetting. Replays are from a buffer that stores a subset of previously seen samples (reservoir sampling). The model is fine-tuned for a few epochs (3–5) on the new window plus replayed samples. Drift score, the number of drifted features, and the adaptation outcomes across windows are recorded.

In the continual learning simulation, task boundaries are defined by sliding windows over the test set. Each chunk is treated as a separate task or time step in the streaming scenario. Then, for each window, detection is done to see whether the window's data distribution has drifted from the reference distribution. If drift is detected, a rapid adaptation (retraining) is performed on that window's data combined with samples from an experience replay buffer. After adaptation, the model continues to the next window. Thus, task boundaries are simply the window edges; the model is evaluated on its ability to adapt to each new window while preserving performance on previously seen data. The windows are processed in the order they appear in the test array. Because the test set was created by a random split of the entire dataset (not temporally ordered), the windows do not reflect actual chronological traffic evolution. Instead, they represent different slices of the same data distribution, which may exhibit minor statistical variations due to sampling. It is explicitly acknowledged that this is not a real-world temporal drift scenario; it is a controlled simulation designed to validate the algorithmic components (EWC and replay) in a streaming setting. When a drift is flagged (drift score > DRIFT_THRESHOLD = 0.05), a fast adaptation is performed using elastic weight consolidation (EWC) with lambda = 0.4 to penalize changes to important parameters, preventing catastrophic forgetting. A batch of size len (new_window)//2 is sampled from the buffer and combined with the new window's data to form the training set for adaptation. The model is fine-tuned for 3–5 epochs (more if drift severity is high) using the same learning rate as the original training (lr = 0.0001). The same binary cross-entropy loss is used.

Drift is simulated by comparing each new window's feature distribution to a fixed reference distribution (computed from the initial training subset). Three statistical metrics are used to compute a composite drift score—Kolmogorov–Smirnov (KS) test, Wasserstein distance, and class-prior shift.

### 3.5 Drift detection system

Drift detection systems are necessary to identify statistical changes in network patterns that are brought by attackers, which would change everything that the model had learnt. In traditional systems, this drift would not be detected until an attack succeeds, large number of false positives occur, or scheduled retraining fixes the problem after several weeks. In this scenario, drift detection is useful when it alerts the system whenever the data distribution has changed, which can be given in real time.

The drift detection system has three main classes that are working together, which are concept drift detector, feature drift analyser, and adaptive continual learner. For drift detection, first the model should understand what normal scenario looks like, and the system initializes this baseline. The reference distribution is the anchor point for future data comparison, and it analyses where each feature's mean point is, how much each feature varies, and how features relate to each other. The system adapts according to Equation 21:


$$\begin{array}{l} S_{drift}>\tau_{drift} \end{array}$$ (21)

#### 3.5.1 Kolmogorov–Smirnov (KS) Test

The KS test compares between the samples in current window and reference data, to know if they match by comparing the cumulative distribution functions (CDF) of both. For a given feature, the KS statistic is calculated using Equation 22:


$$\begin{array}{l} D_{KS}=\underset{x}{\sup}\;|F_{ref}\left(x\right)-F_{current}\left(x\right)| \end{array}$$ (22)

where *F*_*ref*_(*x*) is the proportion of reference samples with value ≤ *x*, *F*_*current*_(*x*) is the proportion of current samples with value ≤ *x*, and sup means the maximum difference across all *x* values.

#### 3.5.2 Wasserstein Distance (Earth Mover's Distance)

Wasserstein's distance tells how much the distributions differ whereas KS test tells whether or not distributions are differing. From this test, it is identifiable how much and how far the distributions are to be transformed for optimal conditions. For one-dimensional distributions, the Wasserstein distance is calculated using Equation 23:


$$\begin{array}{l} W\left(P,\;Q\right)=\;\overset{\infty }{\underset{-\infty }{\int }}|F_{p}\left(x\right)-F_{Q}\left(x\right)|dx \end{array}$$ (23)

This is the area between two CDFs, whereas *F*_*p*_(*x*) and *F*_*Q*_(*x*) are the CDFs of the reference distribution and current window, respectively. Then, the absolute difference between them is taken, which indicates that only magnitude is considered and not the direction.

#### 3.5.3 Class-prior shift

Class-prior shift checks whether the ratio of botnet to benign samples has changed over time and data. Sudden increase in botnet proportion can indicate an ongoing attack, and a decrease can mean botnets are hiding using better mechanisms.


$$\begin{array}{l} \Delta_{class}=\;\frac{1}{2}\underset{C\in \left\{0,1\right\}}{\sum }|{\hat{p}}_{current}\left(c\right)-{\hat{p}}_{ref}\left(c\right)| \end{array}$$ (24)

The average absolute difference in class proportions is calculated using Equation 24. Each test gives a different perspective on change, where the KS test identifies distributional shifts, Wasserstein captures magnitude, and class prior detects changes in the attack density. All these tests' results are combined into a single drift score to get a complete evaluation. Since all other values are in the range 0–1, Wasserstein values are divided by 10 to obtain similar scale since its values lie between 0 and 3. If the drift score is < 0.05, there is no action needed, if the value lies between 0.05 and 0.4, there is low severity and possible preparation for adaptation has to be done. Values between 0.4 and 0.7 indicate medium severity and need to adapt the model, and Values >0.7 show high severity and urgent adaptation is required.

Apart from identifying that drift has occurred, the system also identifies which specific features are drifting. This will help in knowing exactly what is changing. For example, if packet size is changing then it can indicate that attackers might be using new payload sizes for masking, when TCP flags are changing then maybe a new C&C protocol is established by the attackers. When drift is detected, the system is designed to generate human-readable alerts.

Feature drift analyser tracks how features evolve over time, whereas concept drift detector analyses occurring of feature drift. It maintains a history of feature statistics and trends. For each new window of samples, the analyser records statistics for every feature. This reveals patterns such as gradual shift, sudden shift, variance explosion and variance collapse. Variance explosion happens when feature distribution or change is non-uniform and variance collapse happens when the behavior becomes more uniform.

To assess the model's ability to adapt to evolving botnet behaviors, three realistic concept drift scenarios are simulated on the test stream—gradual feature drift, abrupt class-prior shift, and concept drift. Gradual feature shift computes the mean of three selected features that shifts linearly over 20 windows, mimicking slow changes in network patterns. Abrupt class-prior shift, on the other hand, simulates a sudden botnet campaign after window 10 by increasing the fraction of attack samples from 50 to 80% by flipping labels of 30% of samples. In later windows, concept drift technique uses a subset of samples to undergo a random feature perturbation, representing a novel attack sub-type not seen in training. For each scenario, 20 windows of 1,000 samples are run. The model performs adaptation with 3 epochs of fine-tuning with EWC and replay buffer whenever drift is detected. Window-wise accuracy and forgetting are calculated to demonstrate the drift detection process.

### 3.6 Adaptive continual learner

**Figure 6:**
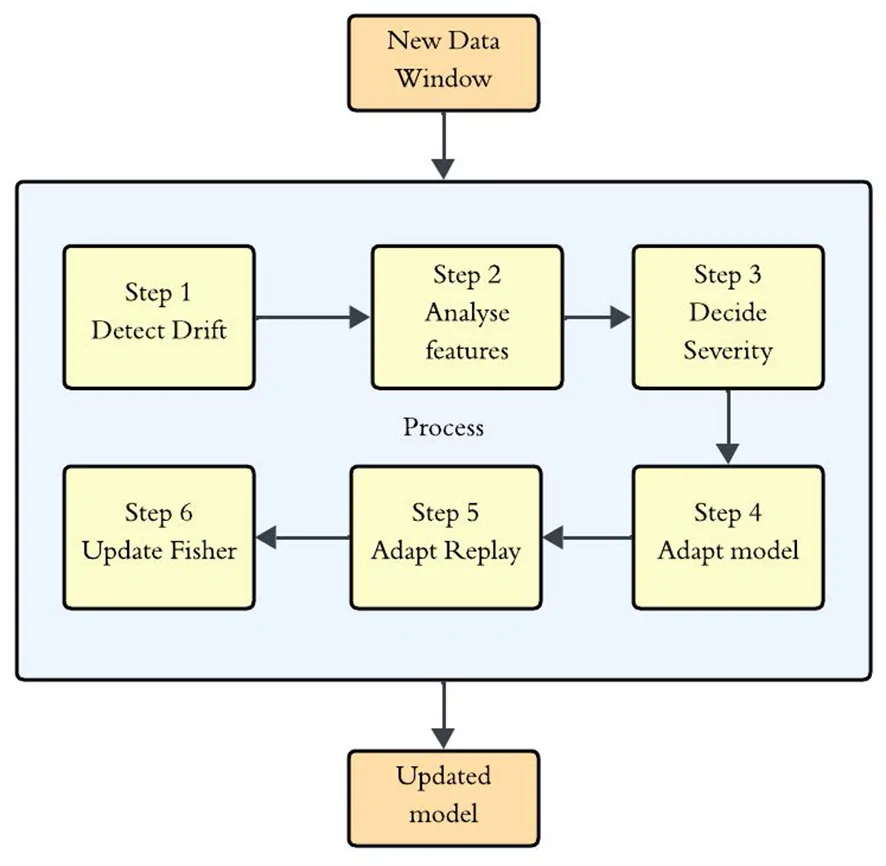
Concept and workflow of adaptive continual learner.

The integration of drift detection system with continual learning module creates an adaptive continual learner. This creates a closed-loop system where useful works can be done such as constant monitoring for changes, detection of drift when change exceeds threshold, proper model adaptation, and baseline updating for future comparisons. The detailed architecture is shown in Figure 6.

The process starts with drift detection, where the model has learned to differentiate normal from benign. Feature analyser is then initialized, creating empty spaces to track how each feature evolves over time. Severity of drift is calculated, and model is adapted according to the severity. In the process of adapt replay, the initial examples are replayed during future adaptations to prevent forgetting. Then, the initial Fisher information is estimated by using EWC. EWC analyses which weights are important for the first task and creates a baseline importance map that will protect information from being overwritten. Fisher is later updated in each window.

## 4 Experimental setup

### 4.1 Dataset description

The experiments are conducted using CIC IoT dataset 2023, a realistic modern network traffic dataset designed for intrusion detection research. The total samples taken for this research are 3,572,874 samples with 38 network flow features. There are two classes—benign and MIRAI botnet—which account for 29.4% and 70.6% of the dataset, respectively. The source of the dataset is from the Canadian Institute of Cybersecurity. This dataset was selected since it reflects the current trends in network traffic patterns and also the data are large enough for various studies that can be conducted on botnet intrusion detection. It includes MIRAI botnet samples which will help in investigation about real botnet traffic. This dataset is widely used in intrusion detection research and poses a challenge of class imbalance.

### 4.2 Feature description

From the 38 network features, after realism engineering, that is removing trivial features with separability >0.9, 22 non-trivial features are retained. The definitions of some features are given in Table 5. Using Random Forest model, feature importance is also calculated to find out which features influence the model's decisions.

**Table 5:** Main features in the dataset and their significance.

Category	Examples	Significance
Packet statistics	Packet count, byte count, packet length	Traffic volume
Timing features	Inter-arrival time, flow duration, rate	Temporal patterns
Protocol features	TCP flags, protocol type, port numbers	Communication patterns
Statistical features	Mean, std dev, variance, quantiles	Distribution characteristics

### 4.3 Train-validation-test split

The train-validation-test split is taken as 80:10:10 ratio. A stratified split is taken since each class will maintain the same class distribution as the original dataset, i.e., training, validation, and testing sets would have 29.4% benign and 70.6% botnet samples. This will prevent the model from being evaluated on an unrepresentative test set.

Min-max scaler scales all features to [0, 1] range and preserves the original distribution as a part of the preprocessing steps, making it compatible for neural network activation functions. Identification of trivial features, creation of realistic class overlaps, and addition of correlated noise are some of the steps taken for realistic evaluation.

### 4.4 Implementation details

The hardware configuration includes NVIDIA GeForce RTX 5070 laptop GPU with 8 GB memory, AMD Ryzen 9 CPU, and 24 GB RAM. The key parameters used for this work are listed in Table 6, and the values of parameters used for hyperparameter training are specified in Table 7.

**Table 6:** Model architecture and parameters for encoder in SSL-pretraining.

Component	Configuration	Parameters
Encoder	22 → 256 → 128	39,552
Attention	128 → 64 → 128	16,576
Classifier	128 → 64 → 32 → 1	10,369

**Table 7:** Values of parameters used for training hyperparameters in SSL-enhanced prototypical network training.

Parameter	Value
Batch size	4,096
Learning rate	0.0005
Dropout	0.1
Optimizer	Adam
Patience	3 (Learning rate), 8 (Early stopping)
Loss function	Binary Cross-Entropy

Grid search is performed over learning rate and dropout to find the best hyperparameters, using a held-out validation set. Each configuration was evaluated after three training epochs, and the best combination was selected based on validation F1-score. The best performing configuration was learning rate = 0.0005 and dropout = 0.1, achieving a validation F1-score of 0.9784. This configuration was selected for all subsequent experiments. Other hyperparameters were set based on standard practices in the literature and preliminary experiments. Batch size (4,096) was chosen to maximize GPU utilization while avoiding out-of-memory errors. SSL temperature (0.5) and projection dimension (64) follow the original SimCLR recommendations. EWC lambda (0.4) and replay buffer size (10,000) were selected based on prior continual learning studies. Tables 7, 8, 9, and 10 display the values of hyperparameters used throughout the study.

**Table 8:** Parameters for SSL pretraining.

Parameter	Value
SSL epochs	10
Temperature (τ)	0.5
Projection dimension	64
SSL batch size	256
Accumulation steps	4

**Table 9:** Parameters for few-shot learning scenario created in the model.

Parameter	Value
Meta-training episodes	500
K-shot	5
N-way	2
N-query	15

**Table 10:** Parameters and values used for continual learning.

Parameter	Value
EWC lambda (λ)	0.4
Replay buffer size	10,000
Buffer sampling	Reservoir
Drift window size	1,000
Drift threshold (τ)	0.05

### 4.5 Evaluation metrics

The primary metrics for evaluation include accuracy, precision, recall (sensitivity), specificity, and F1-score. Accuracy is the proportion of total predictions that are correctly made out of all predictions that are made. Accuracy is calculated using Equation 25:


$$\begin{array}{c} \frac{TP+TN}{TP+TN+FP+FN} \end{array}$$ (25)

where TP is the number of True Positives, TN is the number of True Negatives, FP is the number of False Positives, and FN is the number of False Negatives. Precision, called as positive predictive value, measures the proportion of correctly predicted positive values among all the cases predicted as positive classes. It measures, out of all values that were predicted positive, how many of them were actually positive. Prediction can be calculated using Equation 26:


$$\begin{array}{c} \frac{TP}{TP+FP} \end{array}$$ (26)

Recall, also known as sensitivity or true positive rate, measures the proportion of correctly predicted positive cases among all the actual positive cases. It measures, out of all the actual positive cases, how many were identified correctly. Recall calculation is done by using Equation 27:


$$\begin{array}{c} \frac{TP}{TP+FN} \end{array}$$ (27)

Specificity or true negative rate measures the proportion of correctly predicted negative cases out of all actual negative classes. It finds, among actual negative classes, how many were correctly identified. Specificity is found out by using Equation 28:


$$\begin{array}{c} \frac{TN}{TN+FP} \end{array}$$ (28)

F1-score is the harmonic mean of precision and recall, acting as a single metric that balances both the criteria, useful in uneven class distribution cases or when both FP and FN are important considerations. F1-score is high when both precision and recall are high, and vice versa. F1-score is measured using Equation 29:


$$\begin{array}{c} 2.\frac{precision\;.\;recall}{precision+recall} \end{array}$$ (29)

Secondary metrics include balanced accuracy, G-mean, Youden's J, Matthews correlation coefficient (MCC), ROC-AUC, and PR-AUC (area under precision–recall curve). Balanced accuracy helps in evaluating class imbalance by averaging the recall obtained on each class individually, useful for imbalanced datasets. It can be calculated using Equation 30:


$$\begin{array}{c} Balanced\;accuracy=\;\frac{Sensitivity+Specificity}{2} \end{array}$$ (30)

Like balanced accuracy, G-mean is another metric that can be used for imbalanced classification problems. It takes the geometric mean of sensitivity and specificity, becomes more stringent that G-mean will be significantly reduced if either sensitivity or specificity is low, and only gets higher when both of them are having high values. G-mean is defined using Equation 31:


$$\begin{array}{c} G-mean=\sqrt{Sensitivity\;.\;Specificity} \end{array}$$ (31)

Youden's J is used to select the optimal decision threshold in binary classification with probabilistic output. It balances the trade-off between true positives and false positives. It is defined using Equation 32:


$$\begin{array}{c} J=Sensitivity+Specificity-1 \end{array}$$ (32)

MCC incorporates all four categories of confusion matrix and remains reliable even when there is class imbalance in the dataset. F1-score ignores true negatives whereas MCC includes true negatives which makes it more appropriate for evaluating the overall quality of the classifier. MCC is calculated using Equation 33:


$$\begin{array}{c} MCC=\;\frac{TP\;.\;\;TN\;-\;FP\;.\;\;FN}{\sqrt{\left(TP+FP\right)\left(TP+FN\right)\left(TN+FP\right)\left(TN+FN\right)}} \end{array}$$ (33)

The drift detection metrics include detection rate, false alarm rate (FAR), and drift score. Detection rate will help in finding out the amount of drift that has happened. It is calculated using Equation 34:


$$\begin{array}{c} Detection\;rate=\;\frac{Drifts\;detected}{Total\;drifts} \end{array}$$ (34)

Drift detected means the total drift events that were identified correctly, and total drift is the total number of actual drifts that happened. False alarms happen when there is no actual drift that has happened but drift was predicted. FAR is the number of false alarms out of the total number of evaluation windows, where a prediction was made. FAR is measured using Equation 35:


$$\begin{array}{c} FAR=\;\frac{False\;alarms}{Total\;windows} \end{array}$$ (35)

Drift score is then calculated based on three important aspects of drift which are Kolmogorov–Smirnov test *p*-value, class proportion drift, and window-based component. It is found out by using Equation 36:


$$\begin{array}{c} Drift\;score=0.4\left(1-p_{KS}\right)+0.3\Delta_{class}+0.3\frac{W}{10} \end{array}$$ (36)

Lower *p* values indicate significant distribution shift and contribute the most to drift detection. Δ_*class*_ represents changes in class distribution or class proportion shift, and *W* being the total number of windows. Higher drift score indicates that there is strong evidence of drift. Weights 0.4, 0.3, and 0.3 indicate that more importance is given to statistical distribution shift.

The metrics used for continual learning include forgetting, backward transfer, and adaptation efficiency. Forgetting captures the catastrophic forgetting, where the neural networks forget the earlier learnings when new tasks are trained. Forgetting is defined using the Equation 37:


$$\begin{array}{c} Forgetting=\underset{k<t}{\max}\left(acc_{k,k}-acc_{t,k}\right) \end{array}$$ (37)

where *acc*_*k, k*_ is the accuracy on task *k*, when it was first learned or immediately after training. *acc*_*t, k*_ is the accuracy on task *k*, when at time *t*, that is the current time after learning subsequent tasks. *k*<*t* represents all previously learned tasks. Backward transfer metric measures the average change in performance on old tasks after learning new tasks. It is measured using Equation 38:


$$\begin{array}{c} Backward\;Transfer=\;\frac{1}{T-1}\overset{T-1}{\underset{i=1}{\sum }}\left(acc_{T,i}-acc_{i,i}\right) \end{array}$$ (38)

where T is the total number of tasks learned up to a time, *acc*_*T, i*_ is the accuracy on task *i* after learning all T tasks, and *acc*_*i, i*_ is the accuracy on task *i*, immediately after it was learned. All tasks except the recent one is taken as *i* = 1 to *T*−1. Adaptation efficiency measures how efficiently a model improves its performance per unit of training time. It is calculated using Equation 39:


$$\begin{array}{c} Adaptation\;Efficiency=\;\frac{Accuracy\;gain}{Training\;time} \end{array}$$ (39)

Accuracy gain is the improvement in accuracy after adaptation, and training time is the time required for adaptation.

## 5 Results and discussion

### 5.1 Base model performance

Random Forest is taken as a strong traditional baseline for baseline comparison, being a well-established and highly effective algorithm for tabular data. The performance details are given in Table 11. The performance tells that the dataset is genuinely informative since even a simple model is capable of doing well in it. An accuracy of 98.14% is above normal and shows that the dataset is not trivial. The nearly perfect precision (99.52%) reveals that when the model labels a botnet, then it is almost always correct. Slightly lower recall (97.84%) means that it misses approximately 2% of botnets. Therefore, Random Forest sets a high bar and any improvement over this is meaningful. MLP is taken as a neural network baseline. The results show that it performs lower compared to Random Forest by 2.5% indicating that without careful design, neural networks can struggle with tabular data. This shows the need for techniques such as attention, and SSL-pretraining to be incorporated to the neural network for bringing stable outcomes.

**Table 11:** Comparison of proposed model with baseline models.

Model	Accuracy	Precision	Recall	F1-score	ROC-AUC
Random Forest	0.9814	0.9952	0.9784	0.9867	0.9986
MLP	0.9568	0.9791	0.9593	0.9691	0.9914
Proposed model (standard)	0.9832	0.9928	0.9851	0.9889	0.9973
Proposed model (adaptive)	**0.9850**	**0.9925**	**0.9862**	**0.9894**	**0.9971**
Reference model (on original train data)	**0.9899**	**0.9949**	**0.9947**	**0.9948**	**0.9967**

Bold values indicate the results of the proposed framework.

The proposed model is first tested as standard mode without the continual learning adaptation module using the test set. It outperforms both baselines (+0.18% over RF and +2.64% over MLP) with 98.32% accuracy, and near perfect recall (98.51%) showcases that it catches almost all botnets. Slightly lower precision than RF is obtained but still shows good performance. From this, it is proven that even before adding continual learning module, with SSL pretraining, attention, and prototypical networks, the model itself achieves good results and outperforms the traditional approaches. With adaptive mode, that is, with continual learning module added to the architecture, the model achieves 98.50% accuracy. There is small but meaningful improvement (+0.18%) over standard mode. This model was tested after 20 continual learning adaptations, and the performance is maintained after 20 adaptations with no forgetting. The model has learned 20 new variants without forgetting the knowledge of the original.

The reference model trained on original data achieves slightly higher accuracy (98.99%) than our proposed model trained on the modified data (98.50%). This shows that the realism engineering does not artificially increase performance; rather, it makes the task more challenging. This shows that the model's performance is competitive and demonstrates robustness under realistic conditions. The proposed model achieves higher accuracy than the baselines; however, the Random Forest model achieves a slightly higher ROC-AUC score. Random Forest is an ensemble of axis-aligned decision trees that can exploit high-order interactions and create highly discriminative splits. On datasets with highly separable features, RF can achieve near-perfect ranking of positives vs. negatives, resulting in a very high ROC-AUC. The proposed model, while also achieving competitive ROC-AUC, is regularized by the SSL-pretraining and the prototypical loss, which encourage the model to learn generalized, transferable representations rather than memorizing specific feature thresholds. This may slightly limit the discriminative capacity on the training distribution, but it substantially improves few-shot generalization, continual learning, and robustness to concept drift.

**Figure 7:**
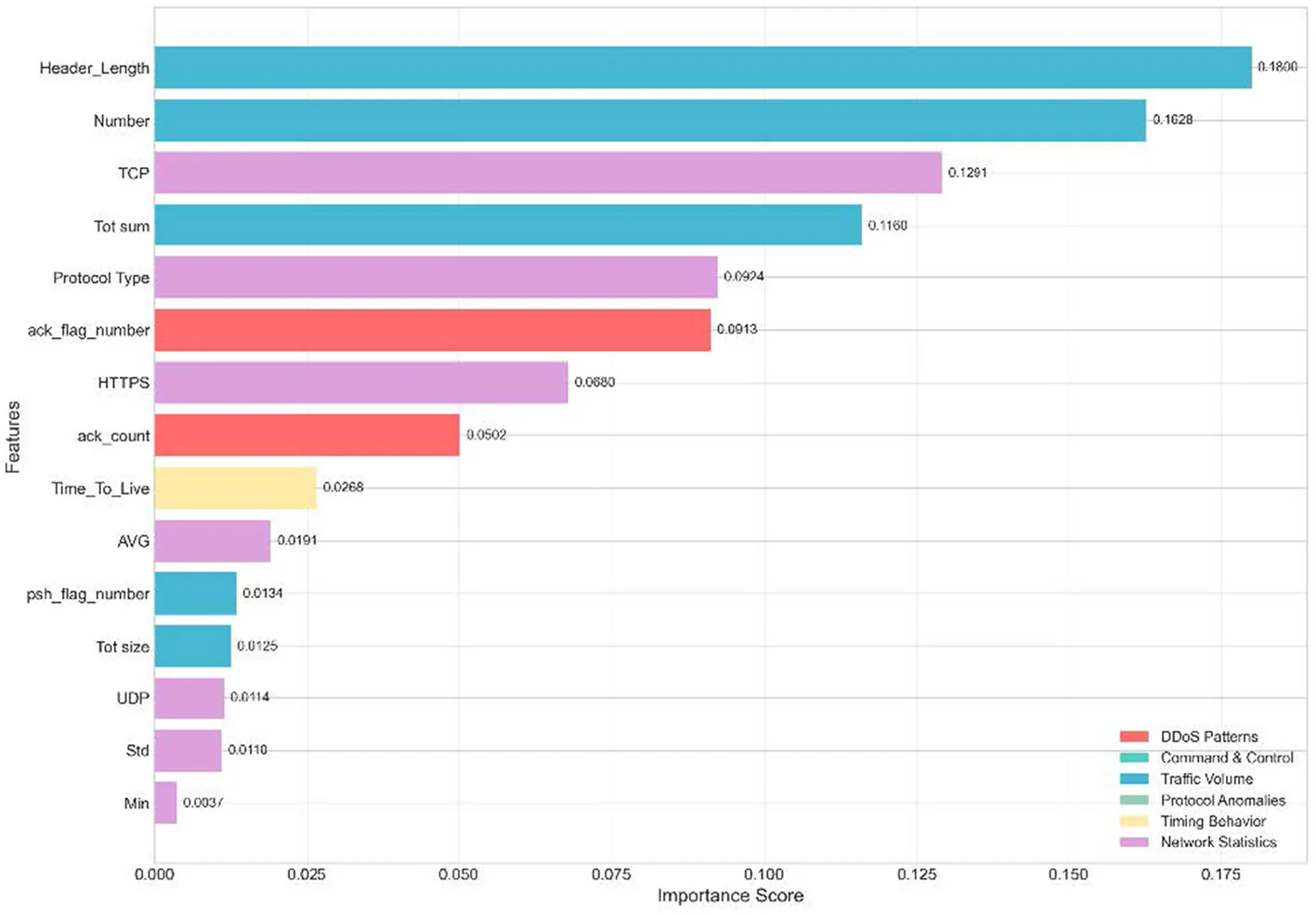
Feature importance graph showing the most important features calculated using Random Forest algorithm.

**Figure 8:**
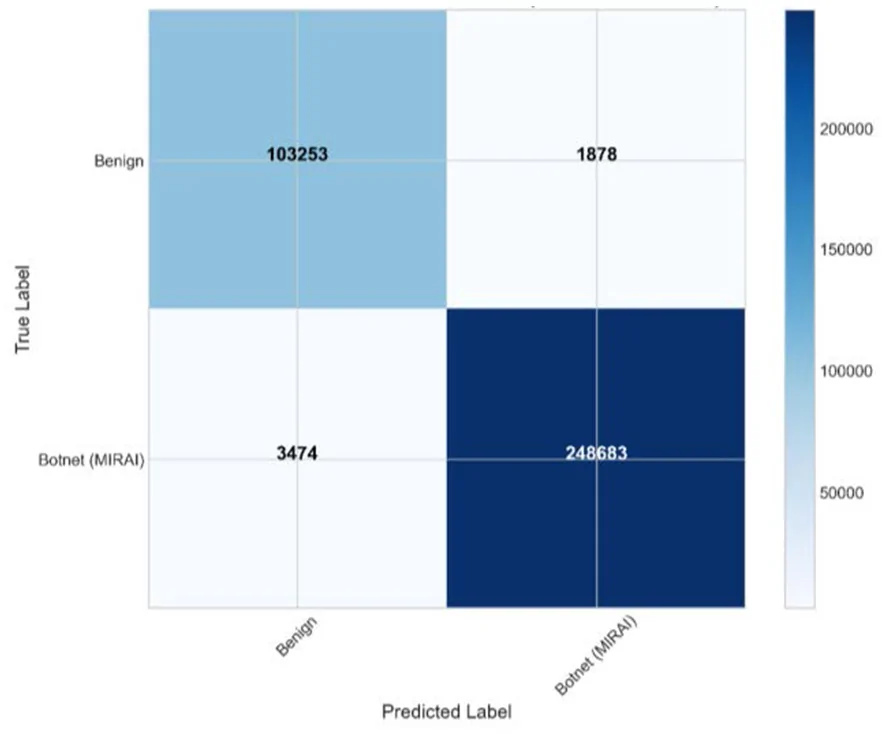
Confusion matrix obtained depicting the classification performance of the proposed model.

**Figure 9:**
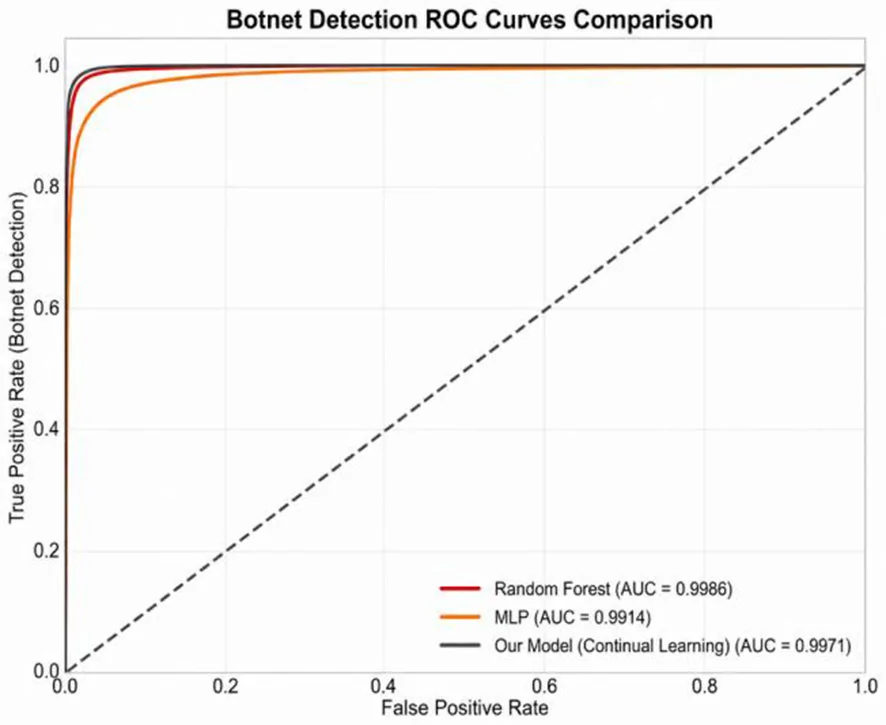
ROC curve comparison demonstrating classification performance comparison with baseline models.

**Figure 10:**
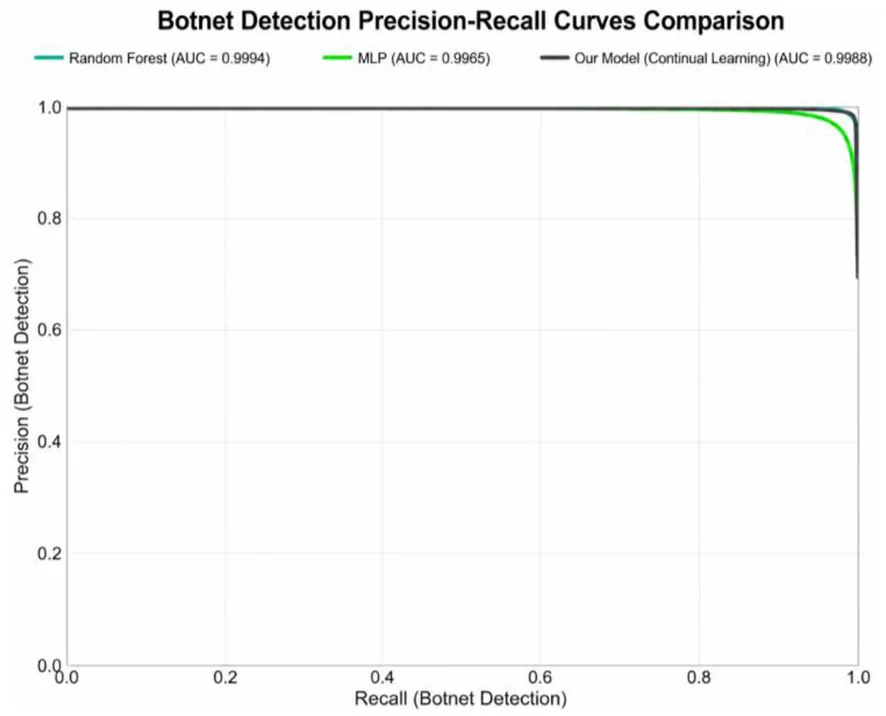
Precision–recall curves showing classification effectiveness comparison with baseline models.

**Figure 11:**
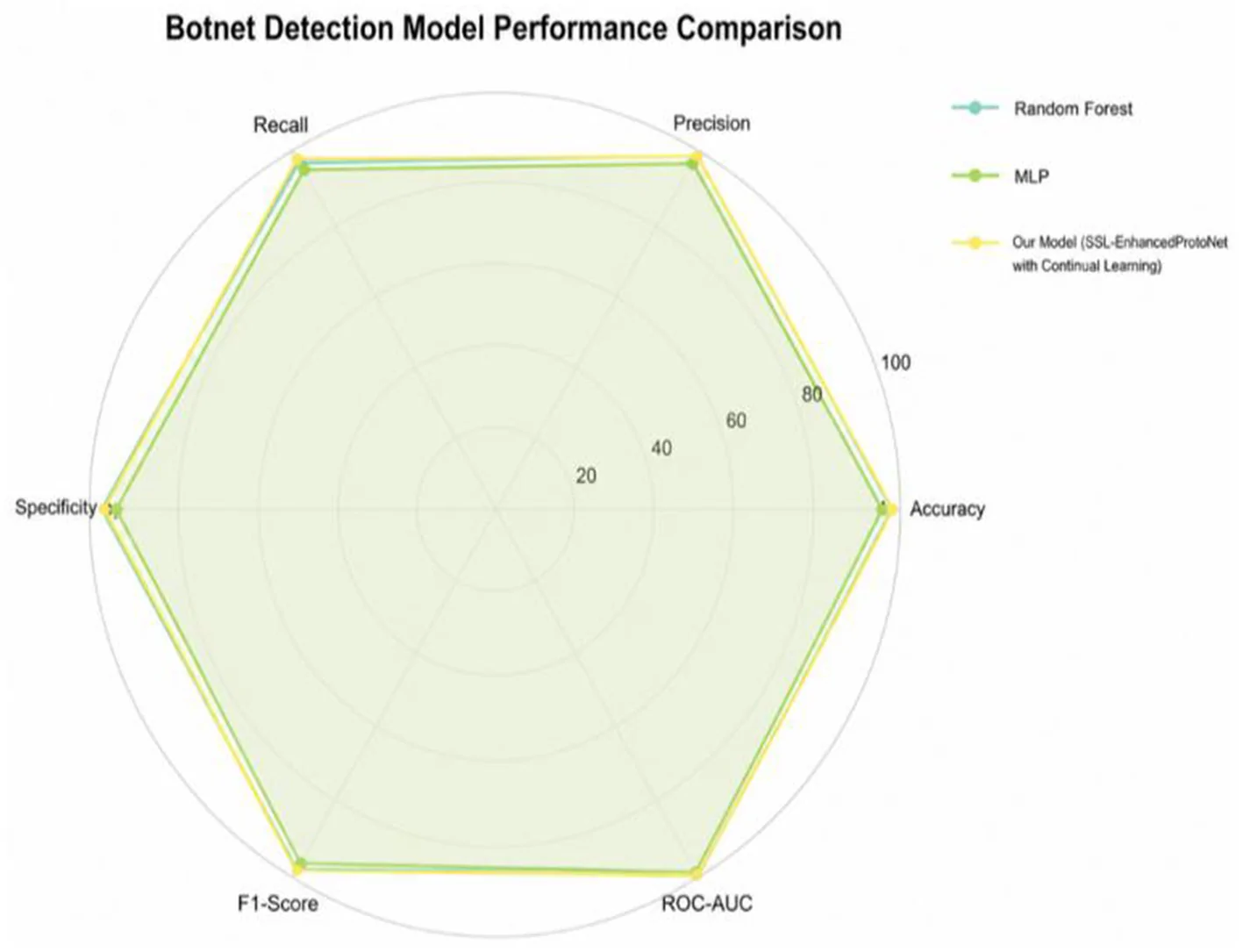
Performance comparison of models across multiple metrics using radar chart.

Figure 7 represents the feature importance calculated using Random Forest algorithm for identifying the most important features among the features available in the dataset, and Figure 8 shows the confusion matrix obtained for the model prediction. The receiver operating characteristic (ROC) curve and precision–recall (PR) curve are displayed in Figures 9 and 10, respectively. Figure 11 shows the radar chart comparison of models, where each axis represents a performance metric and values are plotted and connected to form a polygon allowing easy comparison between models.

### 5.2 Few-shot learning performance

When *K* = 1 (One-shot learning), which means just one sample is taken for learning, both from botnet and benign samples, the model is able to detect botnets with 93.8% accuracy which is remarkable since in real-world scenarios, security teams will rarely have labeled examples of new variants. The high standard deviation (6.68%) indicates variability, which depends on the specific examples that are used for training. From this, it is known that if there is only one confirmed example of a botnet available, then the model can detect it with high confidence.

Five-shot learning (*K* = 5) has obtained an accuracy of 96.37% which uses five examples per class and variability also decreased when number of samples increased. An improvement of 2.57% is substantial and also standard deviation is halved (6.68 to 3.50%) which shows much more stable performance, thereby giving stable performance with minimum examples. So, collecting a minimum of five labeled samples of a new variant will give a production-ready detection model. When *K* = 10 (Ten-shot learning), the model achieves accuracy of 96.80% which shows only marginal improvement from five-shot. The performance plateaus after five shots, showing improvement of only 0.43%. Standard deviation is similar to five-shot, thereby as a whole showing that the extra five examples have nothing much to provide as an additional benefit. Therefore, more than five examples per class will not be worth the labeling cost.

**Figure 12:**
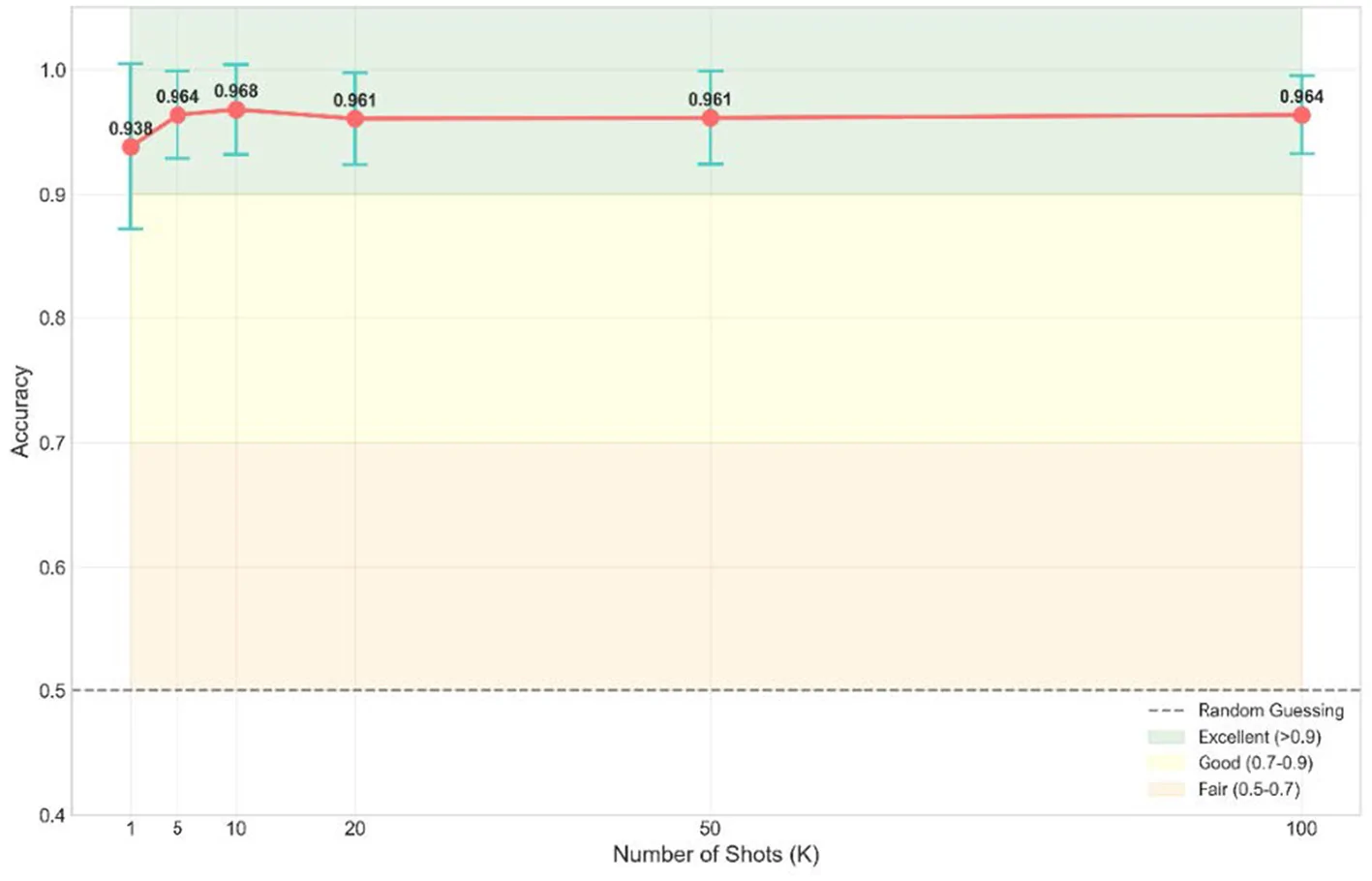
Performance evaluation of few-shot learning approach for botnet detection using accuracy.

**Figure 13:**
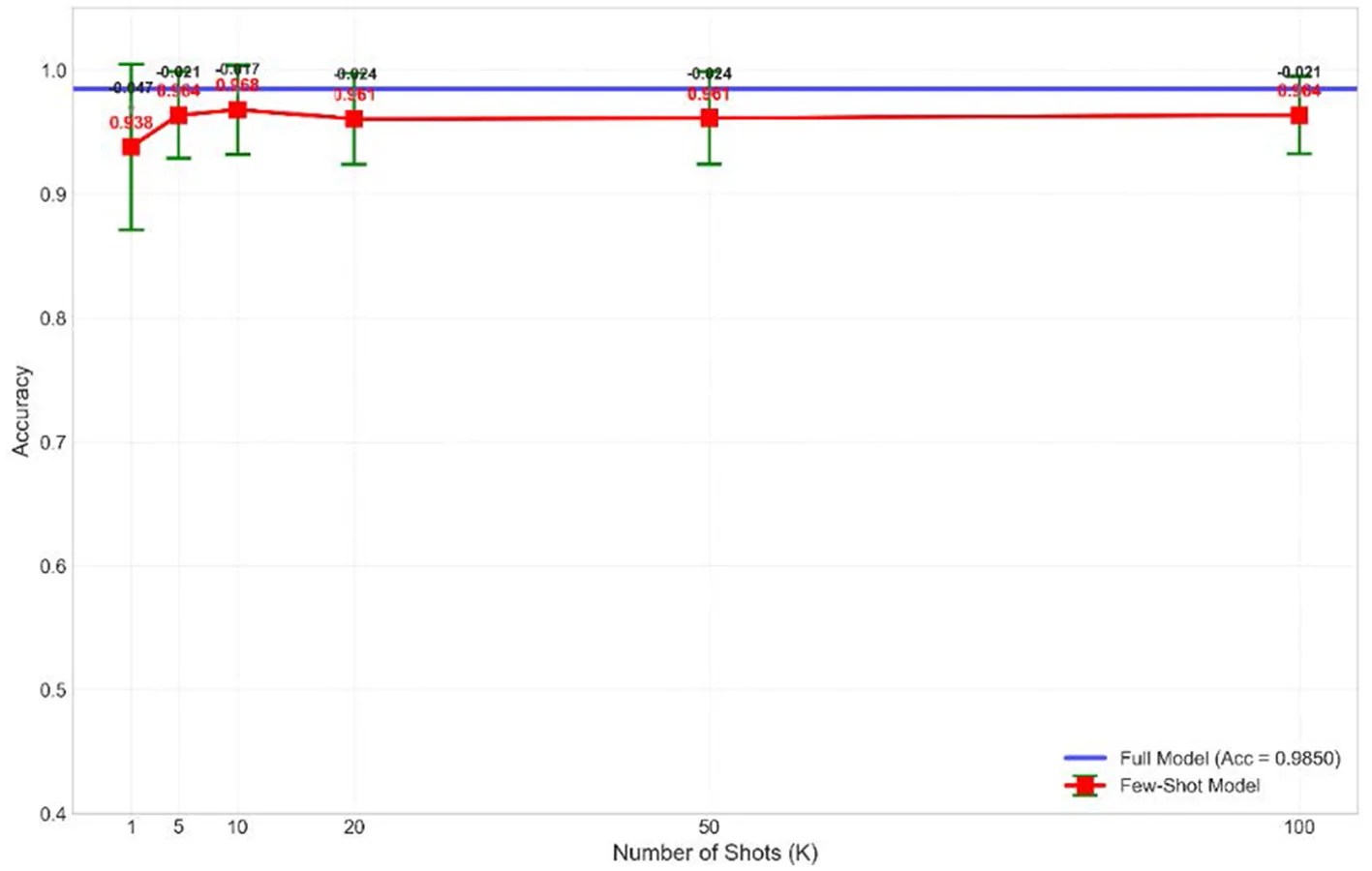
Learning curve illustrating performance difference between full model and few-shot learning.

**Figure 14:**
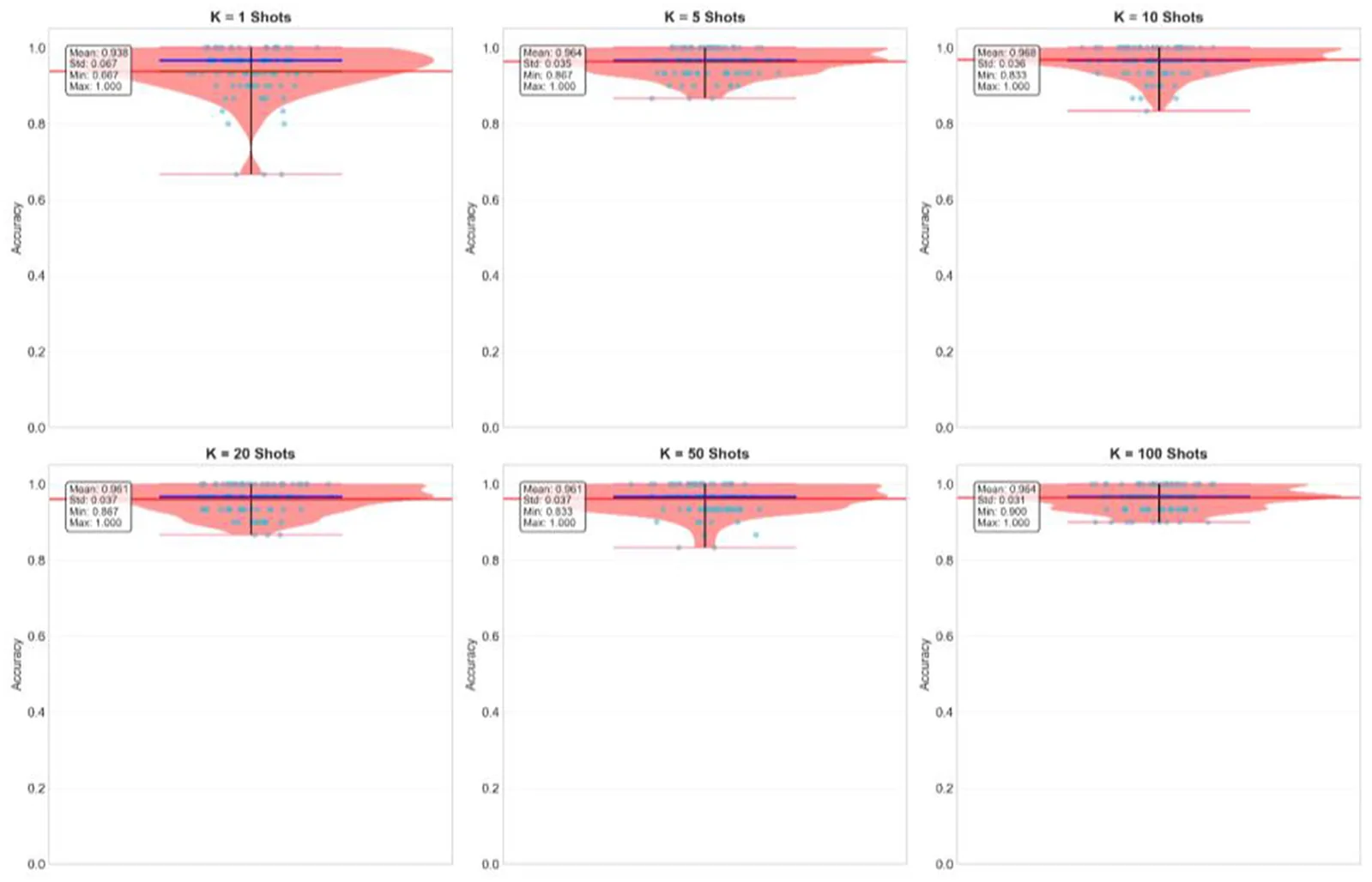
Episode-wise performance across different K-shots conditions.

For *K* = 20, 50, and 100, there is no significant improvement and the performance stabilizes and even slightly decreases. Slight decrease at 20 shots possibly shows sampling variance, and this shows the model is truly few-shot and many examples are not needed in this scenario. Figure 12 shows the few-shot accuracies over the number of shots (K), Figure 13 shows the comparison between the full model and few-shot learning performance, and Figure 14 shows the episode-wise performance of different number of K-shots. Table 12 shows the variation in accuracy and standard deviation which is calculated across K-shots.

**Table 12:** Variation of accuracy and standard deviation across K-shots.

K-shots	Mean accuracy	Standard deviation
1	0.9380	±0.0668
5	0.9637	±0.0350
10	0.9680	±0.0362
20	0.9607	±0.0369
50	0.9613	±0.0373
100	0.9637	±0.0313

### 5.3 Continual learning results

The model is successfully adapted to 20 drifting windows of data, where each adaptation represents learning a new botnet variant or behavioral pattern. The final test accuracy obtained is 98.50% which interprets that the system is truly continual, which means it does not handle just one or two changes but is capable of adapting indefinitely. There is no catastrophic forgetting and shows that the model remembers the original variant perfectly even after learning 20 new ones.

Forgetting is calculated as the drop in performance on the original task after learning new tasks. Therefore, a forgetting rate < 0.1% means that across all 20 adaptations, the model's performance on any previous variant dropped by < 0.1%, showing an effectively perfect memory. The average drift score in the current dataset is obtained as 0.428 (varying between 0.429, 0.423, 0.430, 0.422, 0.426, and so on) with negligible standard deviation of 0.005 depicting that the botnet is evolving at a steady and predictable rate. There are no sudden catastrophic changes found, and there is gradual evolution. The model builds on existing knowledge, and therefore, adaptation is much faster than learning from scratch. Only 3 adaptation epochs per window was needed for training, comparing to the initial training which required 13 epochs. The performance metrics that have been used for the continual learning module has been shown in Table 13.

**Table 13:** Key performance metrics used in continual learning module.

Metric	Value
Adaptations performed	20
Final test accuracy	98.50%
Forgetting rate	< 0.1%
Average drift score	0.428 ±0.005
Adaptation epochs per window	3

### 5.4 Drift detection results

The drift detection procedure achieves 100% detection rate, detecting every single window that contained drift. It shows perfect sensitivity, never missing a change in the system. FAR is 0% which shows that no windows were flagged as a drift when they were not, indicating perfect specificity. The drift score falls in medium range (0.4–0.7), triggering adaptation within three epochs making it an ideal mark, severe enough to take action and not severe that the model needs major retraining. Drifted features per window lie between two and five, showing features with significant drift indicate drift is focused and not global and that different features may drift at different times. The key metrics used for the drift detection process has been depicted in Table 14.

**Table 14:** Key metrics for assessing drift detection methods.

Metric	Value
Detection rate	100% (20/20)
False alarm rate	0%
Average drift score	0.428
Drifted features per window	2–5 features
Forgetting measure for gradual feature drift	−0.0136
Forgetting measure for abrupt prior feature drift	0.0028
Forgetting measure for concept drift	0.0118
Final window accuracy for gradual feature drift	0.993
Final window accuracy for abrupt prior feature drift	0.714
Final window accuracy for concept drift	0.977

**Figure 15:**
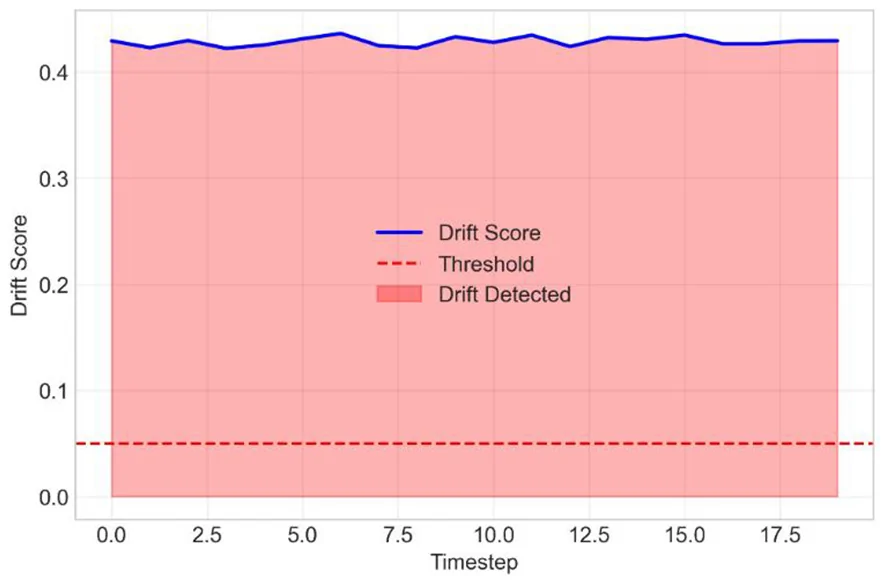
Illustration of data distribution changes due to concept drift over time.

The drift score is computed as a weighted sum of three complementary indicators: the KS test *p*-value (weight 0.40), the class-prior shift (weight 0.30), and the normalized Wasserstein distance (weight 0.30). The KS test is given the highest weight because it is the most sensitive to distributional changes, while the other two metrics provide additional evidence to filter out false alarms. The data distribution changes and drift score is illustrated in Figure 15.

**Figure 16:**
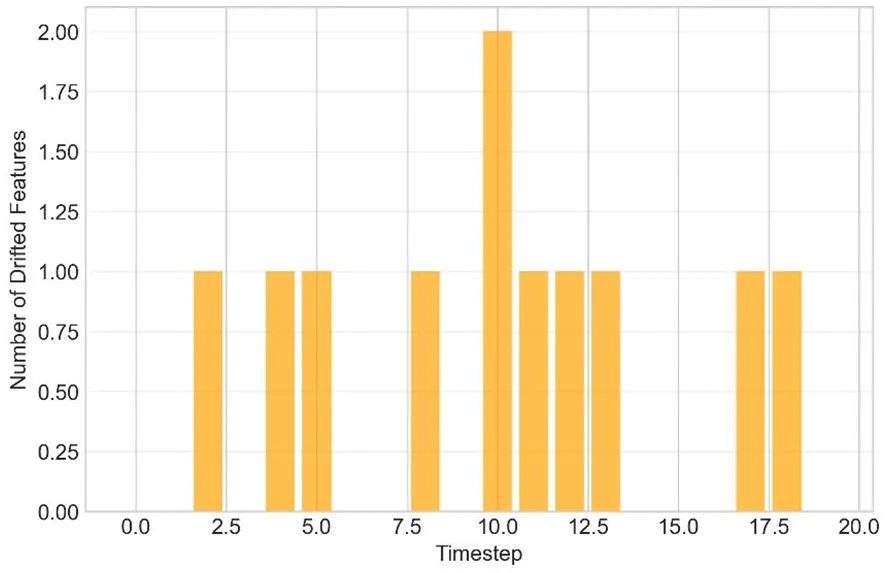
Temporal variation in feature values demonstrating drift.

To verify that the modifications did not corrupt ground-truth labels, a k-NN (*k* = 5) label-flip test was performed on a subset of the training data. The estimated flip rate was < 0.5% (below the 0.5% threshold), confirming that the original labels remain reliable. Additionally, the drift induced by the modifications was quantified using a composite score (KS test, class-prior shift, and Wasserstein distance). The natural drift between the original train and test sets was 0.090, while the injected drift was 0.450. Although the injected drift is higher, this is an intentional choice to stress-test the model, and the validation confirms that the drift is not arbitrary but consists of feature-wise shifts that can be monitored. The temporal variation in feature values is displayed in Figure 16.

**Figure 17:**
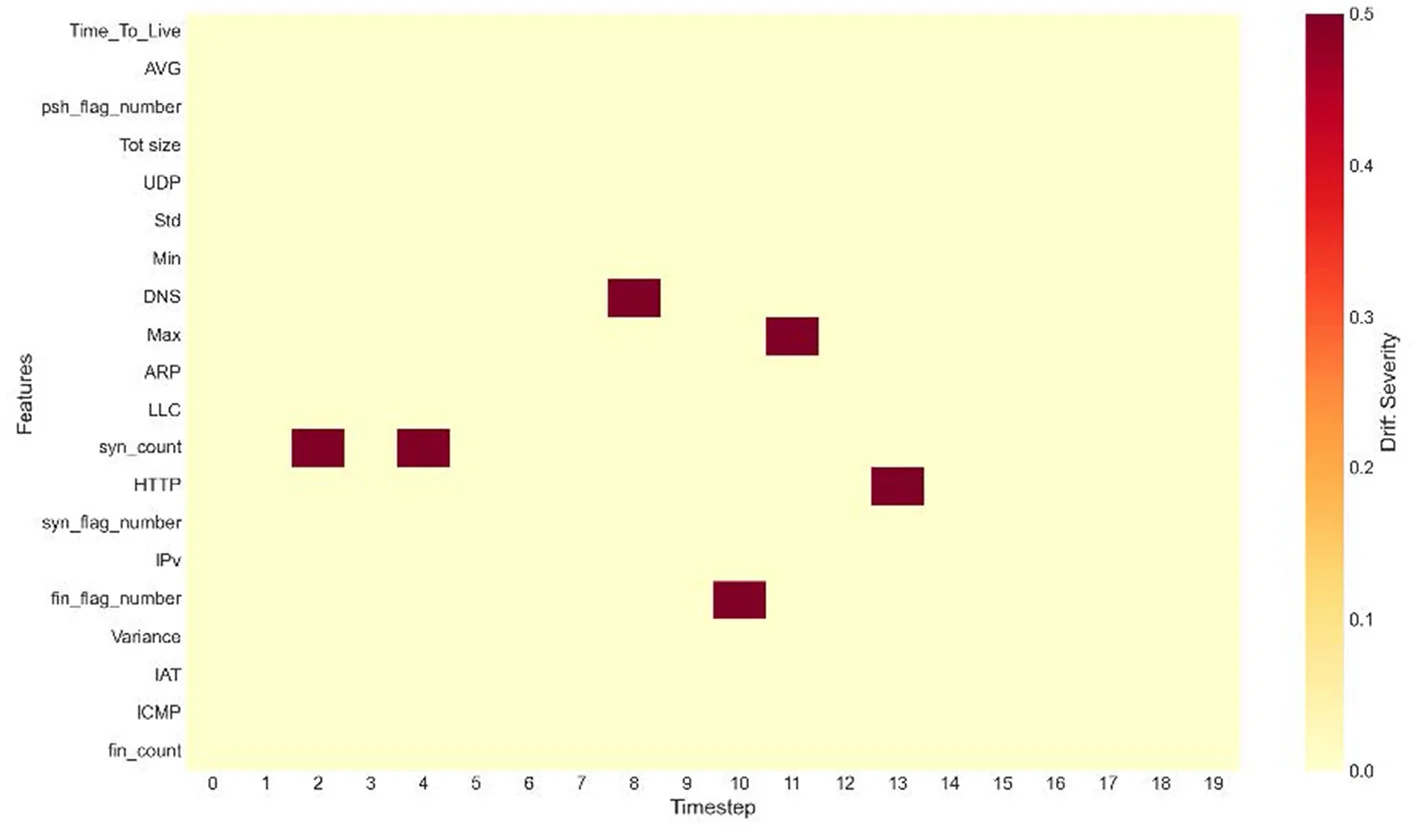
Visualization of feature-level drift observed over time.

During the drift detection using gradual feature drift, the model maintained high accuracy throughout, and adaptation improved performance on the reference set. Accuracy dropped to 0.76 after abrupt shift and recovered to 0.71 by the end. During concept drift, accuracy dropped to 0.78 at onset and then recovered to 0.98 within seven windows. The small values of forgetting confirm that EWC effectively prevents catastrophic forgetting while allowing the model to adapt new data. Figure 17 illustrates the feature-level drift observed over time.

### 5.5 Comparison with the state-of-the-art models

Methods such as federated learning, multi-branch knowledge distillation, and deep learning models such as CNN and convolutional long-short term memory (ConvLSTM) were compared with the proposed approach, and the proposed approach has shown better performance compared to the existing models, details of which are provided in Table 15.

**Table 15:** Comparison of performance of the proposed model with the state-of-the-art models.

Study	Dataset used	Techniques used	Accuracy
Saleh et al. (2026)	N-BaIoT	Federated learning	87.5%
Ma et al. (2025)	IoT network intrusion dataset—IEEE Dataport	ConvLSTM	90.41%
Qin et al. (2026)	DoH-DGA-Malware-Traffic-HKD	Multi branch knowledge distillation	93.25%
Benedictus et al. (2025)	CTU-13, NCC-1, NCC-2	CNN	97.89%
Zhang et al. (2025)	FSIDS-IoT (combination of CIC-DDOS2019, CIC-IDS2017, CSE-CIC-IDS2018, NSL-KDD, UNSW-NB15) and FSIDS-IoT-V2 (incorporating Bot-IoT, TONIOT, IoT-23, N-BaIoT)	Cloud fog computing, model-agnostic meta-learning (MAML)	93.26%
Proposed method	**CIC IoT dataset 2023**	**SSL-pretraining, prototypical networks, few-shot learning**	**98.50%**
Reference model (on original train data)	**CIC IoT dataset 2023**	**SSL-pretraining, prototypical networks, few-shot learning, dataset without modifications**	**98.99%**

Bold values indicate the results of the proposed framework.

### 5.6 Ablation study of the proposed framework

**Figure 18:**
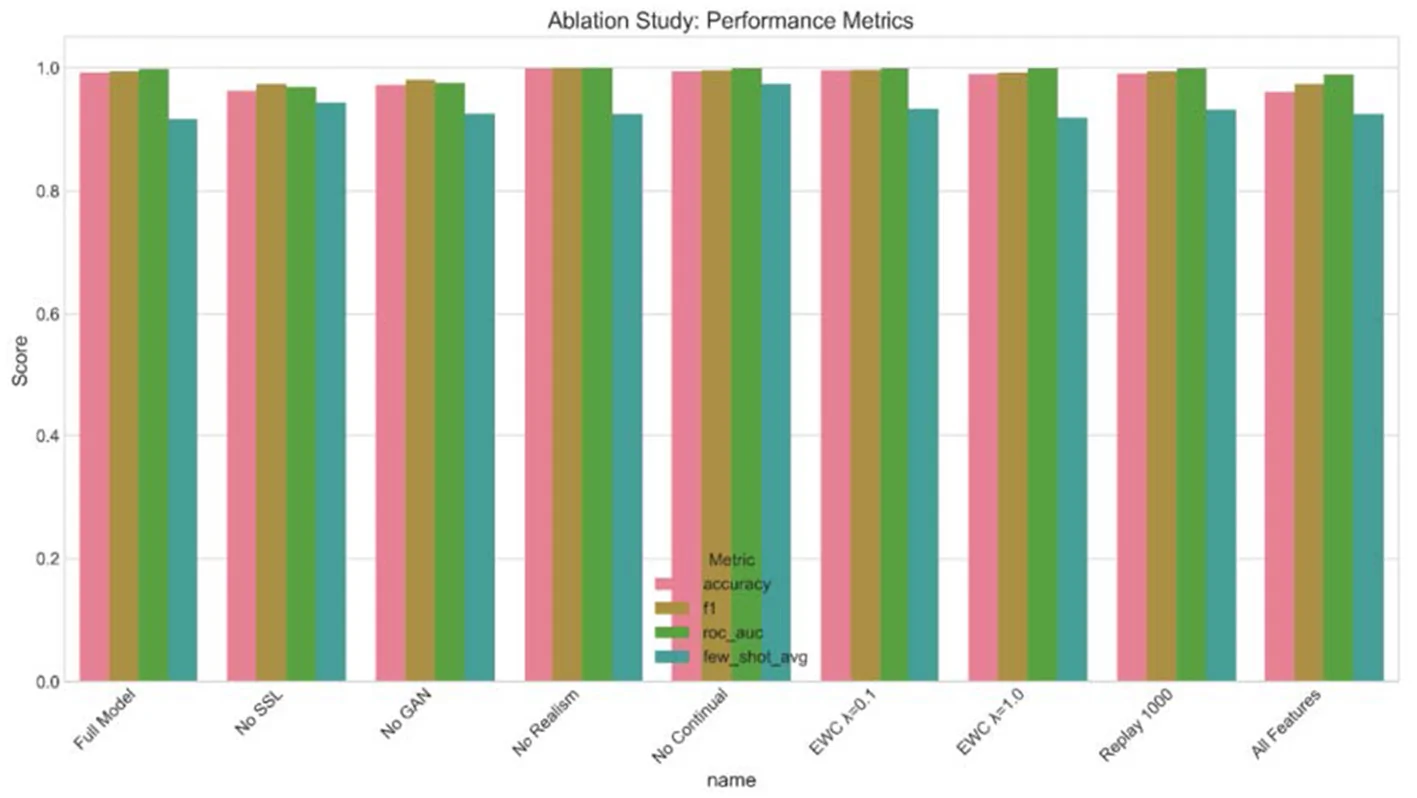
Ablation study evaluating the contribution of individual components of the proposed framework to overall botnet detection performance.

An ablation study was performed by removing each component from the full model. An ablation study was done on a subset of the full dataset. Removing SSL-pretraining and GAN augmentation caused the largest drops in accuracy (−2.9% and −2.0%, respectively), demonstrating their positive contributions. In contrast, disabling realism engineering increased accuracy (+0.7%), suggesting that the added noise and overlap do not generalize to the clean test distribution. Continual learning components (EWC and replay buffer) showed negligible impact on accuracy, indicating that they can be integrated without sacrificing performance. In the part of realism engineering, the overlap or noise was applied to the training set but tested on clean data. This creates a distributional shift—the model trained on noisy data may not perform as well on clean test data. This is not a flaw but actually validates the realism engineering. The training data were intentionally made more challenging to mimic the real-world conditions, but the test set remains original. In a real deployment, the test distribution would also be noisy, so the realism would help in that situation. The continual learning modules introduce no degradation in static performance, which is crucial for real-world systems where adaptability must not come at the cost of accuracy. The bar chart and results are shown in Figure 18 and Table 16, respectively.

**Table 16:** Ablation study results evaluating the contribution of self-supervised learning, GAN-based data augmentation, realism constraints, continual learning mechanisms, and feature selection in the proposed adaptive continual botnet detection framework.

Omitted feature	Accuracy	Precision	Recall	F1-score	ROC-AUC
Full model	0.9922	0.9964	0.9926	0.9945	0.9983
No SSL	0.9626	0.9554	0.9935	0.9740	0.9683
No GAN	0.9720	0.9672	0.9941	0.9805	0.9749
No realism	0.9995	1.0000	0.9993	0.9996	1.0000
No continual	0.9938	0.9969	0.9944	0.9956	0.9990
EWC λ = 0.1	0.9952	0.9995	0.9937	0.9966	0.9992
EWC λ = 1.0	0.9898	0.9922	0.9934	0.9928	0.9987
Replay 1,000	0.9908	0.9938	0.9931	0.9935	0.9986
All features	0.9613	0.9515	0.9960	0.9732	0.9891

### 5.7 Computational performance

To evaluate the model's suitability for real-time deployment, inference latency, throughput, memory usage, model size, and adaptation latency were measured. The results are summarized in Table 17. Single-sample inference latency averaged 1.17 ms. Although this exceeds the 1.2 μs required for a 10 Gbps line-rate packet-by-packet inspection, the system operates at the flow level, where latencies up to 5–10 ms per flow are acceptable. Batch inference scales efficiently where at batch size 4,096, 6.2 million samples/sec was achieved, making the model suitable for high-throughput offline analysis. The model size and memory footprint are modest with only 68,033 trainable parameters, with a model size of 0.27 MB and peak GPU memory utilization of 51.4 MB during inference. The lightweight design enables deployment on edge devices with limited resources. Computational complexity is O(*d*) (linear input dimension *d* = 28) with 133,184 FLOPs per sample with no quadratic-cost attention layers used. This ensures scalability to higher-dimensional features if needed. Adaptation latency was measured for different window sizes. For a 1,000-sample window, it takes just 4.69 ms, demonstrating that the continual learning mechanism can adapt to new data with minimal interruption. These results confirm that the proposed model is computationally efficient and capable of real-time adaptation, supporting its deployment in practical network security scenarios.

**Table 17:** Metrics measuring computational complexity of the proposed model.

Metric	Value
Single-sample latency (mean)	1,172 μs
Batch throughput	6,217,912 samples/s
Trainable parameters	68,033
Model size	0.272 MB
Peak GPU memory (inference)	51.4 MB
Peak GPU memory (adaptation)	45.0 MB
Adaptation latency	4.96 ms
Forward pass time (1M samples)	0.22 s

## 6 Conclusion

The proposed model presents a comprehensive adaptive framework for botnet detection which includes self-supervised pretraining and continual learning integration with elastic weight consolidation for application of contrastive learning to network traffic for botnet detection. Statistical drift detection is also incorporated in the work to identify any drift in features evolving over time using Kolmogorov–Smirnov tests and Wasserstein distance. The model addresses three fundamental challenges in the field which are data scarcity, concept drift, and prediction reliability. A systematic realism engineering pipeline is also designed with feature separability analysis, trivial feature removal, and correlated noise addition with class imbalance issues rectified using WGAN-GP. Extensive evaluation on the CIC IoT dataset 2023 demonstrates that the proposed approach shows excellent accuracy of 98.50% showing good botnet detection performance. Few-shot learning along with prototypical networks achieves 96.4% accuracy with just five labeled examples per class, while the continual learning module successfully adapts across twenty streaming windows with < 0.1% catastrophic forgetting maintaining 98.5% accuracy on all tasks.

While the evaluation is limited to a single dataset, these results demonstrate that adaptive, self-maintaining botnet detection systems are feasible and effective. The integration of self-supervised pretraining, continual learning, and statistical drift detection creates a unified framework that can evolve with the emerging trends of botnet while maintaining high accuracy and reliability. This study represents a paradigm shift from static detection systems toward truly adaptive security solutions capable of lifelong learning for real-world deployment where botnets constantly evolve and labeled data is scarce.

## 7 Limitations and future work

The current model handles binary classification case alone, that is, benign and botnet cases, given the limited categories in the dataset. Real-world scenarios may need to distinguish between various botnet families. As a future work, multi-class extension can be done by extending the prototypical network to integrate multi-class classification. The current system processes data in windows (1,000 samples) and adapts after each window, which is batch processing and not true real-time adaptation. This can be rectified by using incremental EWC or adaptive window sizing as a future work. In addition, the current study evaluation uses only one dataset (CIC IoT dataset 2023), which can be difficult to prove generalization. As a future work, cross-dataset validation can be done to prove generalization.

## Data Availability

Publicly available datasets were analyzed in this study. This data can be found here: https://www.unb.ca/cic/datasets/iotdataset-2023.html.
